# Decoding monocyte signatures in ischemic stroke: A multi-scale transcriptomic approach

**DOI:** 10.4103/NRR.NRR-D-24-01669

**Published:** 2025-09-03

**Authors:** Yanyi Peng, Bo Xiao, Mengqi Zhang

**Affiliations:** 1 Department of Neurology, Xiangya Hospital, Central South University, Changsha, Hunan Province, China; 2 National Clinical Research Center for Geriatric Disorders, Xiangya Hospital, Central South University, Changsha, Hunan Province, China

**Keywords:** abhydrolase domain-containing protein 2, bulk-RNA sequencing, CellChat, diagnosis model, gene signature, ischemic stroke, monocytes, phagocytosis, reactive oxygen species, single-cell transcriptomics

## Abstract

Monocytes play a crucial role in post-stroke immune infiltration, yet the intricate immune regulatory networks they orchestrate in ischemic stroke remain poorly understood. This knowledge gap has hindered the development of targeted monocyte-based therapies for stroke. Here, we used a multi-omics approach combining single-cell and bulk transcriptomics. CellChat analysis revealed intercellular communication networks, while key genes were identified and predictive models built through Lasso regression. Immune cell infiltration dynamics were quantified using single-sample gene set enrichment analysis. Gene set enrichment analysis and gene set variation analysis identified disease-regulated pathways of core genes. MicroRNA networks and transcription factors were investigated using mircode and RcisTarget. Experimental validation was performed using oxygen–glucose deprivation and transient middle cerebral artery occlusion models, focusing on the influence of abhydrolase domain-containing protein 2 on monocyte function. We observed significantly elevated monocyte content in stroke brain tissue samples, and identified key monocyte genes associated with immune inflammation, chemokine signaling, and cell receptor function. A robust seven-gene predictive model for ischemic stroke was developed. CD274 strongly correlated with these seven genes, suggesting a potential immunomodulatory axis. *In vivo* transient middle cerebral artery occlusion experiments validated the predictive value of key genes. *In vitro* studies demonstrated that abhydrolase domain-containing protein 2 overexpression enhanced monocyte proliferation and phagocytic activity post-oxygen–glucose deprivation while reducing reactive oxygen species generation. In conclusion, this study maps post-stroke monocyte communication networks, identifies key signaling pathways, identifies regulatory mechanisms, and validates the functional importance of key genes, particularly abhydrolase domain-containing protein 2. These findings provide a foundation for developing targeted immunomodulatory therapies and precision diagnostics in ischemic stroke management.

## Graphical abstract

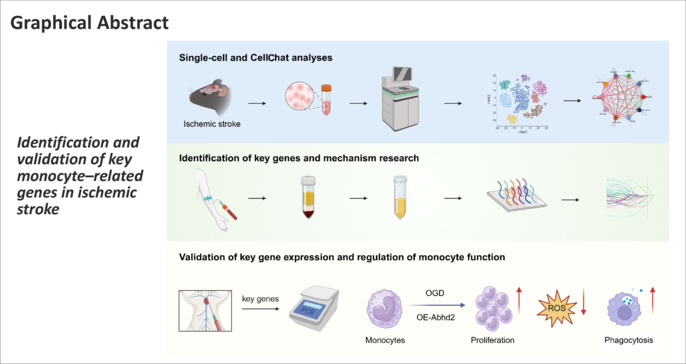

## Introduction

Ischemic stroke is the predominant stroke subtype, accounting for approximately 87% of all cases (Tsao et al., 2022), and underscoring the urgent need for innovative approaches in its early diagnosis, effective treatment, and prevention. The pathophysiology of stroke is intricate, involving a complex interplay of neuroglial cells, immune cells, neurons, vascular components, and extracellular matrix elements. While hemodynamic abnormalities and vascular pathology are critical (Campbell et al., 2019), mounting evidence highlights the pivotal role of immune and inflammatory responses in stroke pathogenesis (Ajoolabady et al., 2021; Qin et al., 2022). The dual nature of immune regulation in ischemic stroke presents both challenges and opportunities. This necessitates comprehensive understanding of the relationship between immune inflammation and cerebral ischemia to unlock new therapeutic avenues. Current standard treatments for ischemic stroke, including intravenous thrombolysis (Xiong et al., 2022; Tsivgoulis et al., 2023) and mechanical thrombectomy (Campbell et al., 2019; Qin et al., 2022; Widimsky et al., 2023), while effective, have limitations. The persistent lack of suitable pharmacological interventions for many stroke patients underscores the critical need to identify and exploit novel therapeutic targets. This gap in treatment options emphasizes the importance of exploring innovative approaches, particularly those targeting the immune–inflammatory axis, to improve outcomes in ischemic stroke.

Ischemic stroke poses a risk of causing irreversible damage to neural networks, which can lead to functional impairments. The immune–inflammatory response following stroke plays a dual role in both injury progression and subsequent repair processes. In response to ischemic damage, innate and adaptive immune reactions in the brain and peripheral tissues actively participate in neural repair after stroke (Liu et al., 2020). Inflammatory signals triggered by immune mediators swiftly activate resident cells, facilitating the infiltration of diverse inflammatory cells into the ischemic area (Jayaraj et al., 2019). This may potentially exacerbate post-ischemic cerebral damage by augmenting blood–brain barrier permeability, edema, and progressive neuronal demise (Pluta et al., 2021). The targeting of inflammatory cytokines and pharmacological interventions at the acute phase of stroke holds therapeutic prospects, yet clinical translation continues to present challenges (Lambertsen et al., 2019). Stroke not only elicits morphological changes in brain cells (Qin et al., 2022; Hu et al., 2025a, b), but also alters the immunological structure of the brain (Li et al., 2022; Zheng et al., 2025). After stroke, there is a noticeable activation of bone marrow hematopoietic stem cells, resulting in increased presence of monocytic cells within the brain (Courties et al., 2015; Ritzel et al., 2015). Following neutrophils, monocytes are notably among the first blood-derived immune cells to infiltrate ischemic tissue (Jayaraj et al., 2019). Monocytes infiltrate brain tissue during the acute phase of stroke (Jurcau and Simion, 2021) and serve as the primary phagocytes at 72 hours after stroke (Ritzel et al., 2015). Inflammatory processes following stroke emerge as both a tool and direction for immunotherapy. However, the mechanisms and targets of monocyte regulation in ischemic stroke still remains unclear. Yet this exploration can provide potential strategies for identifying immune therapeutic targets in stroke and establish a theoretical foundation for the translation of anti-inflammatory therapies in stroke treatment.

The heterogeneity, temporal dynamics, and interactions of cellular subpopulations in brain tissue following stroke have not been fully clarified. This knowledge gap has restricted the development of precisely targeted therapeutic strategies for specific cells. Recent advancements in single-cell ribonucleic acid sequencing (scRNA-seq) have revolutionized our ability to dissect cellular heterogeneity and understand complex molecular networks in ischemic stroke. ScRNA-seq enables the analysis of cell cluster heterogeneity post-stroke at single-cell resolution (Qiu et al., 2022). This allows the identification and characterization of stroke-associated cell subclusters. Therefore, we have approached our research from a single-cell perspective, specifically focusing on monocytes using transcriptomic sequencing data from peripheral blood mononuclear cells to further analyze and explore the biological significance of key disease-related genes in ischemic stroke. We then further investigated the functional roles of key genes in disease progression and the molecular regulatory mechanisms by simulating ischemic stroke conditions *in vivo* and *in vitro* through animal models and cell experiments. By determining the primary molecular drivers and cellular communication networks in ischemic stroke, our work sets the stage for targeted interventions designed to enhance clinical outcomes in stroke management.

## Methods

### Data acquisition

The study used publicly available gene expression data from the Gene Expression Omnibus (GEO), a repository hosted by the National Center for Biotechnology Information (NCBI). Specific datasets were selected for analysis to encompass both single-cell and bulk transcriptomic profiles relevant to ischemic stroke. “Ischemic stroke” and “single cell” were used as keywords to select appropriate single-cell sequencing datasets in the GEO database (https://www.ncbi.nlm.nih.gov/geo/). Then “ischemic stroke” was used as the keyword to screen suitable transcriptome profiles, with “expression profiling by high throughput sequencing” and “Homo sapiens” as limiting conditions. The primary dataset for single-cell analysis, GSE174574 (https://www.ncbi.nlm.nih.gov/geo/query/acc.cgi?acc=GSE174574), comprised brain tissue scRNA-seq data from six male C57BL/6J mice at 6–8 weeks of age. This included a middle cerebral artery occlusion (MCAO) group (24 hours) and sham group (three mice per group). This dataset was chosen for its detailed single-cell resolution, enabling insights into the cellular heterogeneity of ischemic stroke pathology. Additionally, the datasets GSE197731 (ipsilateral *vs.* contralateral hemispheres in MCAO-1d and MCAO-2d mice), GSE210986 (sham *vs.* MCAO-3d mice), and GSE142445 (ipsilateral *vs.* contralateral hemispheres in MCAO-4h and MCAO-7d mice) were systematically investigated for time-dependent changes in monocytes following MCAO. Subsequently, bulk transcriptomic data were incorporated from GSE22255 and GSE16561 to broaden the scope of gene expression analysis and enhance robustness of the study findings. GSE22255 (annotated under platform GPL570) includes 40 sample sets, evenly divided between 20 healthy controls and 20 patients with ischemic stroke. Similarly, GSE16561 (under platform GPL6883) includes 63 sample sets with 24 controls and 39 stroke patients. The datasets used in this study, along with detailed information, can be found in **Additional Table 1**.

**Additional Table 1:** Summary of dataset information

GEO accession	Platform	Data type	Model	Organism/strain	Sex	Age	Tissue	Number of samples in control group	Number of samples in disease group
GSE174574	GPL21103	scRNA-seq	MCAO	C57BL/6 mice	Male	60-70 d	Brain	3 (1d sham)	3 (1d_MCAO)
GSE142445	GPL24247	scRNA-seq	MCAO	C57BL/6 mice	Male	6 wk	Brain	2: GSM4227433 (4h_contralateral), GSM4227439 (7d_contralateral)	2:GSM4227434 (4h ipsilateral), GSM4227440 (7d ipsilateral)
GSE197731	GPL17021	scRNA-seq	MCAO	C57BL/6 mice	Male	8-9 wk	Brain	GSM5929213 (1d_contralateral), GSM5929217 (2d_contralateral)	GSM5929214(1 d_ips ilateral), GSM5929218 (2d ipsilateral)
GSE210986	GPL24247	scRNA-seq	MCAO	C57BL/6 mice	Male	/	Brain	1 (3d sham)	1 (3d_MCAO)
GSE22255	GPL570	Bulk RNA-seq	/	Homo sapiens	Female 20 Male 20	45-74 yr	Peripheral blood Mononuclear cells	20	20
GSE16561	GPL6883	Bulk RNA-seq	/	Homo sapiens	Female 36 Male 27	≥18 yr	Blood	24	39
GSE37587	GPL6883	Bulk RNA-seq	/	Homo sapiens	Female 40 Male 28	≥18 yr	Blood	/	68
GSE124026	GPL5175	Bulk RNA-seq	/	Homo sapiens	/	>18 yr	Blood	/	11

### Single-cell analysis

Expression profiles of GSE174574 were processed using the Seurat package (https://satijalab.org/seurat/). This involved filtering out genes with low expression (nFeature_RNA > 50 & percent.mt < 25), followed by data standardization, normalization, and principal component analysis (PCA). The optimal number of principal components was determined to be 17 by elbow plot analysis. Spatial relationships among clusters were visualized using uniform manifold approximation and projection (UMAP). Cell types crucial in disease processes were annotated using the celldex package, and marker genes for each cell subtype were identified using the FindAllMarkers function, with significant markers defined by |avg_log2FC| > 1 and p_val_adj < 0.05. Cell subtypes were annotated using the R package, SingleR. For Seurat objects created using GSE197731, GSE210986, and GSE142445, cells meeting the criteria of 200 < nFeature_RNA < 6500, mitochondrial percentage < 40%, and hemoglobin percentage < 3% were retained for further analysis.

### Ligand–receptor interaction analysis

CellChat (https://github.com/sqjin/CellChat) was used to quantitatively infer and analyze intercellular communication networks from single-cell data. It employs network analysis and pattern recognition techniques to predict the primary signal inputs and outputs of cells, as well as the modes of coordination between these cells and signals. In this analysis, standardized single-cell expression profiles serve as input data, providing cellular information to aggregate communication probabilities and compute the intercellular communication network. This calculation explored cell-related interactions, quantifying the strength (weights) and frequency (count) of interactions between cells to observe the activity levels and influence of each cell type in the context of disease. The cells were divided into sham and MCAO groups to identify major signal changes under different biological conditions. Subsequently, the most frequently communicating cells were selected for further analysis.

### Model construction

To develop a predictive model for ischemic stroke, a multi-step approach was taken. Candidate genes were initially identified based on their potential relevance to stroke pathophysiology. Subsequently, least absolute shrinkage and selection operator (Lasso) regression was used to construct predictive models, leveraging its ability for simultaneous variable selection and regularization in high-dimensional datasets. For each patient, a risk score was calculated by integrating expression values of selected genes, weighted by their respective Lasso-derived coefficients: Risk Score = Σ(β_i × Expression_i), where β_i represents the Lasso-estimated coefficient for the i-th gene. The model formula was: RiskScore = SERPINH1 × (–0.0823132474259442) + TCF4 × (–0.0582012978640228) + ABHD2 × (–0.0451857418404853) + PLAC8 × (–0.0109059063134779) + GNGT2 × (–0.00853274369014924) + CDH5 0.00924752635136245 + SLC22A8 × 0.0688908952798146. Patients were stratified into low-risk and high-risk groups using the median risk score as the dichotomization threshold. The model’s discriminative performance was evaluated by receiver operating characteristic curve analysis, with the area under the curve (AUC) quantified as a measure of predictive accuracy.

### Immune cell infiltration analysis

To examine the impact of genes on immune cell infiltration, a single sample gene set enrichment analysis algorithm was used to quantify the levels of immune cell infiltration in each sample. The “ggplot2” package (https://ggplot2.tidyverse.org/) was used to visualize the relative abundance of immune cells in each sample, where the sum of scores for all estimated immune cell types within each sample equaled 1. Interactions among immune cells were analyzed and illustrated by a heatmap generated using the “corrplot” package (https://cran.r-project.org/web/packages/corrplot/index.html) to highlight correlations among different immune cell types. Violin plots were produced to display the relative abundance of immune cells. The Wilcoxon test was used to calculate differences in immune cell content, thereby providing insights into the impact of genes on immune infiltration. Spearman correlation analyses were used to explore relationships between gene expression levels and immune cell content.

### Development trajectory of key cell subtypes

Single-cell studies facilitate the delineation of transcriptional regulation in intricate physiological processes and highly diverse cell populations. These investigations aid the discovery of genes specific to particular cell subtypes, genes that demarcate intermediate states in biological processes, and genes implicated in transitions between distinct cell fates. In numerous single-cell analyses, gene expression processes are engaged asynchronously by cells, with each cell capturing a snapshot of the ongoing transcriptional dynamics. Monocle is used to introduce a strategy for ordering individual cells along pseudotime. This leverages their asynchronous processes to position them on trajectories correlating with biological processes such as cell differentiation.

### Gene set enrichment analysis

Gene set enrichment analysis (GSEA) was performed using predefined gene sets to rank genes according to differential expression patterns between two sample groups. Enrichment of these gene sets at the extremes (top or bottom) of the ranked list was then statistically evaluated. In this study, GSEA was used to compare differences in signaling pathways between the high-expression group and low-expression group, aiming to investigate the molecular mechanisms of core genes in both patient groups. The permutation number was set to 1000, and the permutation type was specified as “phenotype.”

### Gene set variation analysis

Gene set variation analysis (GSVA) is a non-parametric and unsupervised approach, which was used to examine the enrichment of gene sets within transcriptomic data. A comprehensive score for the gene sets of interest was generated by GSVA, which transformed gene-level variations into pathway-level alterations, facilitating examination of the biological functions of samples. To mitigate interference from redundant pathway information, duplicate genes within each gene set and genes appearing in two or more pathways were excluded. Gene sets comprising 50 hallmark pathways were obtained from the Molecular Signatures Database (https://software.broadinstitute.org/cancer/software/gsea/wiki/index.php/MSigDB_v7.0_Release_Notes). Subsequently, the GSVA algorithm, available in the R programming package “GSVA” (https://www.bioconductor.org/packages/release/bioc/html/GSVA.html), was used to comprehensively score each gene set. This facilitated the assessment of potential variations in the biological functions of different samples.

### MicroRNA network construction

MicroRNAs (miRNAs) are a class of small non-coding RNAs that regulate gene expression by either promoting the degradation of mRNAs or inhibiting mRNA translation. Hence, the aim was to determine whether certain miRNAs within key genes regulate the transcription or degradation of specific risk genes. After obtaining miRNAs associated with key genes from the mircode database (http://www.mircode.org/), the miRNA network of genes was visualized using Cytoscape software (https://cytoscape.org/).

### Regulatory network analysis of model genes

The R package “RcisTarget” (https://www.bioconductor.org/packages/release/bioc/html/RcisTarget.html) was used to predict transcription factors. All computations executed by RcisTarget were based on motifs. The normalized enrichment score (NES) of motifs depended on the total number of motifs in the database. In addition to motifs annotated by source data, further inference of annotation files was made based on motif similarity and gene sequences.

The first step was to estimate overexpression of each motif on the gene set by calculating the AUC for each motif–gene set pair. This calculation was determined from recovery curves based on the ranking of motifs by gene sets. The NES for each motif was computed based on the distribution of AUCs for all motifs in the gene set. Subsequently, the “rcistarget.hg19.motifdb.cisbpont.500bp” database was used for ranking gene motifs.

### Molecular docking

The three-dimensional (3D) protein structures of key genes were retrieved from the PDB database (https://www.rcsb.org/) and AlphaFold database (https://alphafold.com/). The CTD database (https://ctdbase.org/) was used to predict interactions between key genes and drugs, with pertinent compounds obtained. Subsequently, drug compound structures were accessed from the PubChem database (https://pubchem.ncbi.nlm.nih.gov/). Molecular docking was conducted using AutoDock software (https://autodock.scripps.edu/), with the genetic algorithm employed as the docking method and 50 docking iterations performed. The docking outcome with the lowest binding energy was identified, and the results imported into PYMOL (https://pymol.org/) for visualization. This showcased the binding sites between small molecules and proteins.

### Establishment of transient focal cerebral ischemia model

Estrogen has been extensively shown to have a protective effect in experimental stroke (Hurn and Brass, 2003). In female mice, estrogen levels fluctuate with the estrous cycle, which could potentially impact experimental outcomes. Moreover, the sequencing data employed in this study were all derived from male C57BL/6 mice. In order to align with prior research and avoid the effects of cyclical hormone fluctuations, male C57BL/6 mice were used in the animal experiments. Transient focal cerebral ischemia was induced in male C57BL/6 mice (8–10 weeks old, 25–28 g) using the MCAO method. Mice were housed under temperature-controlled conditions with a 12-hour light/dark cycle and free access to food and water. A 1-week acclimation period was allowed before the experiments. The experiments were approved by the Animal Care Committee of Xiangya Hospital, Central South University (approval No. 201906718, approval date June 17, 2019) and conducted in strict accordance with the National Institutes of Health Guide for the Care and Use of Laboratory Animals (8^th^ ed., National Research Council, 2011). The study was reported in accordance with ARRIVE guidelines (Kilkenny et al., 2010). Mice were anesthetized by inhalation with isoflurane (3% induction, 1.5 % maintenance; RWD Life Science Co., Ltd., China) delivered in 30% O_2_/70% N_2_O. A midline cervical incision was made to expose the right common carotid artery, internal carotid artery, and external carotid artery (ECA). The distal portion of the ECA was ligated, and a nylon monofilament was inserted into the internal carotid artery via the ECA and advanced until it blocked the origin of the middle cerebral artery. Laser Doppler flowmetry was used to monitor regional cerebral blood flow and confirm a decrease to < 25% of baseline levels. The occlusion was maintained for 60 minutes, after which the filament was withdrawn to allow reperfusion. Mice in the sham group underwent the same surgical procedure without filament insertion. Animals that did not meet the regional cerebral blood flow reduction criteria or did not survive the surgery were excluded from the study. Five mice were assigned to each of the experimental (MCAO) and control (sham) groups. The MCAO group underwent 24 hours of reperfusion following the 60-minute occlusion.

### Monocyte isolation

Mice were deeply anesthetized by inhalation with isoflurane (5% for induction until loss of pedal reflex, 2% for maintenance; RWD Life Science Co., Ltd.) delivered in a carrier-gas mixture of 30% O_2_ and 70% N_2_O. Peripheral blood was collected by cardiac puncture into ethylenediaminetetraacetic acid-coated tubes. The anticoagulated blood was mixed gently with an equal volume of physiological saline. The diluted blood was then slowly added to an equal volume of Ficoll-Paque PLUS (Cytiva, Logan, UT, USA) in a 15 mL centrifuge tube. The tube was centrifuged at 800 × *g* for 30 minutes at room temperature. After centrifugation, the mononuclear cell layer was carefully aspirated and transferred to another 15 mL centrifuge tube, resuspended in 10 mL of physiological saline, and centrifuged at 400 × *g* for 5 minutes at 4°C. The supernatant was discarded and the cell pellet resuspended in 10 mL of RPMI-1640 medium (glucose, amino acids, and L-glutamine, HyClone, Cytiva, Cat# SH30027.01). This washing step was repeated once more. Finally, the cell pellet was resuspended in 2–3 mL of RPMI-1640 medium.

### Flow cytometric analysis of CD14^+^ monocytes

To characterize isolated monocytes, cells were stained with FITC-conjugated anti-mouse CD14 monoclonal antibody (Reddot Biotech Inc., Kelowna, BC, Canada, Cat# RD21930F) for 30 minutes at 4°C. After washing with phosphate-buffered saline, cells were analyzed using a BD FACSCalibur flow cytometer (BD Biosciences, San Jose, CA, USA). Data analysis was performed using FlowJo software (https://www.bdbiosciences.com/en-us/products/software/flowjo-v10-software). The percentage of CD14^+^ monocytes within the isolated cell population was determined.

### Quantitative reverse transcription-polymerase chain reaction

For the MCAO model, monocytes were isolated from five mice per group (MCAO and sham) at 24 hours post-reperfusion. Total RNA was extracted from isolated monocytes using TRIzol reagent (Invitrogen, Carlsbad, CA, USA) according to the manufacturer’s protocol. The quantity and quality of the extracted RNA were assessed using a NanoDrop spectrophotometer (Thermo Fisher Scientific, Waltham, MA, USA). First-strand cDNA synthesis was performed using the iScript cDNA Synthesis Kit (Bio-Rad, Hercules, CA, USA) following the manufacturer’s instructions. Quantitative reverse transcription-polymerase chain reaction (qRT-PCR) was performed using iTaq Universal SYBR Green Supermix (Bio-Rad) on a CFX Connect Real-Time PCR Detection System (Bio-Rad). For the amplification of target genes, gene-specific primers were designed and synthesized by Shanghai Sangon Biotech: serine peptidase inhibitor, clade H, member 1 (*Serpinh1*), transcription factor 4 (*Tcf4*), abhydrolase domain-containing protein 2 (*Abhd2*), placenta-specific 8 (*Plac8*), guanine nucleotide binding protein (G protein), gamma transducing activity polypeptide 2 (*Gngt2*), cadherin 5 (*Cdh5*), and solute carrier family 22 member 8 (*Slc22a8*). The housekeeping gene, glyceraldehyde-3-phosphate dehydrogenase (*Gapdh*), served as an internal control for normalization. The primer sequences are shown in **Table 1**.

**Table 1:** The primer sequences used for quantitative reverse transcription-polymerase chain reaction

Primer	Sequences (5'–3')	
	Forward	Reverse
*Serpinh1*	GCT GAG AAG CTG CGC GAT GA	AGG CGG CTG CCC AGT TTC CA
*Tcf4*	CGG GAT GAA TCA GCC CGG CT	AGA CTG GAG TTG ATG TCT GC
*Abhd2*	CAC TTC TAC CTT CGA CCT CT	TCA ACG AAG GTT CGG ATA TA
*Plac8*	CCA GCC TGT GTG ATT GCT TC	CCA CAC AGA CAA CAC TCA TT
*Gngt2*	AAG GAG CTG TTG AGG ATG GA	TCT ACA TAA TCC TTG ATT TC
*Cdh5*	ATT GGC CTG TGT TTT CGC AC	GAT TTG GTA CAA GAC AGT GG
*Slc22a8*	AGC CAG TGG CTC CAG TGC TG	CCA CAT TCA AGA TAA TGG TG
*Gapdh*	GAT GCC CCC ATG TTT GTG AT	CAG GGG GGC TAA GCA GTT GG

*Abhd2*: Abhydrolase domain-containing protein 2; *Cdh5*: cadherin 5; Gapdh: glyceraldehyde-3-phosphate dehydrogenase; *Gngt2*: guanine nucleotide binding protein (G protein), gamma transducing activity polypeptide 2; *Plac8*: placenta-specific 8; *Serpinh1*: serine peptidase inhibitor, clade H, member 1; *Slc22a8*: solute carrier family 22 member 8; *Tcf4*: transcription factor 4.

For *in vitro* studies, monocytes isolated from healthy mice were subjected to either oxygen–glucose deprivation (OGD) treatment or *Abhd2* overexpression. For OGD experiments, monocytes were exposed to OGD conditions for 0, 12, or 24 hours before RNA extraction. In *Abhd2* overexpression experiments, monocytes were transfected with either the *Abhd2* overexpression plasmid (OE-Abhd2) or the empty vector (OE-NC), with RNA extracted 48 hours post-transfection. Untreated monocytes cultured under normal conditions served as controls. RNA extraction and cDNA synthesis for these *in vitro* experiments followed the same protocol as described for the MCAO model. qRT-PCR was performed specifically for the *Abhd2* gene, with *Gapdh* serving as the housekeeping gene.

### Cell culture and oxygen–glucose deprivation treatment

Monocytes were isolated from the peripheral blood of healthy C57BL/6 mice (8–10 weeks old) using the method described above. These monocytes were used for *in vitro* experiments to simulate ischemic conditions and study the effects of Abhd2 overexpression. Mouse monocytes were isolated from peripheral blood and cultured in RPMI-1640 medium (HyClone, Cat# SH30027.01) supplemented with 10% fetal bovine serum (Hyclone, Cat# SH30087.01) and 1% penicillin/streptomycin at 37°C in a humidified atmosphere containing 5% CO_2_. For OGD treatment, cells were washed twice with phosphate-buffered saline and cultured in glucose-free RPMI-1640 medium (Gibco) in a hypoxic chamber (1% O_2_, 5% CO_2_, and 94% N_2_) for 0, 12, or 24 hours. Control cells were maintained in normal RPMI-1640 medium under normoxic conditions.

### Transfection of the Abhd2 overexpression plasmid

The coding sequence of mouse Abhd2 was synthesized and cloned into the pcDNA3.1 vector (General Biosystems Co., Ltd., Anhui, China). Monocytes were seeded in 24-well plates at a density of 2 × 10^5^ cells/well and cultured in RPMI-1640 medium containing 10% fetal bovine serum. When the cells reached the desired density, they were transfected with the OE-Abhd2 or OE-NC using Lipofectamine 2000 (Invitrogen) according to the manufacturer’s instructions. Briefly, 1 μL of Lipofectamine 2000 and 0.5 μg of plasmid DNA were diluted separately in 50 μL of Opti-MEM (Invitrogen), incubated for 5 minutes at room temperature, and then combined and incubated for an additional 20 minutes. The transfection complex was added to each well containing cells in Opti-MEM. After 5 hours, the medium was replaced with fresh complete medium. Cells transfected with OE-Abhd2 or OE-NC were subjected to OGD treatment for 24 hours before subsequent experiments. Transfection efficiency was confirmed by qRT-PCR. All experiments were performed with at least three biological replicates.

### Western blot analysis

Monocytes subjected to different durations of OGD treatment (0, 12, and 24 hours) were lysed in radioimmunoprecipitation assay buffer containing protease inhibitors. Protein concentrations were determined using the bicinchoninic acid method. Equal amounts of protein were separated by sodium dodecyl sulfate–polyacrylamide gel electrophoresis (Bio-Rad Laboratories, Cat# 456-1034) and transferred to polyvinylidene difluoride membranes (Millipore Sigma, Burlington, MA, USA). Membranes were blocked with 5% skim milk and incubated overnight at 4°C with Abhd2 polyclonal antibody (rabbit, 1:1000, Proteintech Group, Rosemont, IL, USA, Cat# 14039-1-AP, RRID: AB_2242089) and anti-GAPDH antibody (rabbit, 1:1000, Cell Signaling Technology, Danvers, MA, USA, Cat# 2118, RRID: AB_561053). After washing, membranes were incubated with horseradish peroxidase-conjugated secondary antibodies (goat, 1:5000, Jackson ImmunoResearch Laboratories, West Grove, PA, USA, Cat# 111035144, RRID: AB_2307391) for 1 hour at 37°C. Protein bands were visualized using ECL Western Blotting Substrate (Thermo Fisher Scientific) and exposed on BioMax XAR X-ray film (Kodak Health Imaging, Rochester, NY, USA) in a darkroom. Relative protein expression was normalized to GAPDH.

### Fluorescent microsphere phagocytosis assay

OGD-treated monocytes (OGD-Cell), monocytes transfected with empty vector (OGD-OE-NC), and monocytes overexpressing Abhd2 (OGD-OE-Abhd2) (General Biosystems Co., Ltd.) were seeded in 24-well plates. They were incubated with Fluoresbrite YG carboxylate microspheres (1 μm, Polysciences, Warrington, PA, USA, Cat# 15702) at a final concentration of 1 × 10^10^ particles/L for 48 hours. Cells were washed with phosphate-buffered saline to remove non-ingested microspheres, stained with 4′,6-diamidino-2-phenylindole (Sigma-Aldrich, St. Louis, MO, USA, Cat# D9542), and imaged using a Leica DMI6000B inverted fluorescence microscope (Leica Microsystems, Wetzlar, Germany). Mean fluorescence intensity of engulfed particles was calculated to assess phagocytosis from three independent experiments.

### 3-(4,5-Dimethylthiazol-2-yl)-5-(3-carboxymethoxyphenyl)-2-(4-sulfophenyl)-2H-tetrazolium cell proliferation assay

At 24 hours post-OGD, OGD-Cell, OGD-OE-NC, and OGD-OE-Abhd2 monocytes were seeded in 96-well plates at a density of 1 × 10^4^ cells/well and cultured under normal conditions for 0, 24, 48, or 72 hours. Cell proliferation was assessed using the CellTiter 96 AQueous One Solution Cell Proliferation Assay (3-[4,5-dimethylthiazol-2-yl]-5-[3-carboxymethoxyphenyl]-2-[4-sulfophenyl]-2H-tetrazolium [MTS], Promega, Madison, WI, USA, Cat# G3582) according to the manufacturer’s protocol. Briefly, 10 μL of MTS reagent was added to each well containing 100 μL of culture medium, and cells were incubated for 4 hours at 37°C. Absorbance at 490 nm was measured using a Thermo Multiskan MK3 microplate reader (Thermo Fisher Scientific). The experiment was performed with six replicates for each condition.

### Measurement of reactive oxygen species

Reactive oxygen species (ROS) levels in monocytes were measured using a ROS Assay Kit (Beyotime, Shanghai, China, Cat# S0033S) according to the manufacturer’s instructions. Briefly, OGD-Cell, OGD-OE-NC, and OGD-OE-Abhd2 monocytes (1 × 10^6^ cells/mL) were loaded with 2,7-dichlorodihydrofluorescein diacetate probe, incubated at 37°C for 30 minutes, washed, and analyzed by flow cytometry (BD FACSCalibur). The non-fluorescent 2,7-dichlorodihydrofluorescein diacetate was converted to the highly fluorescent 2,7-dichlorofluorescein upon oxidation by intracellular ROS. The percentage of 2,7-dichlorofluorescein-positive cells, which reflects intracellular ROS levels, was determined by flow cytometry using a BD FACSCalibur instrument. The experiment was performed with three biological replicates, and the data analyzed using FlowJo software.

### Statistical analysis

Data are presented as mean ± standard error of the mean. For comparisons between two groups, Student’s *t*-test was used. For multiple group comparisons, one-way analysis of variance followed by Tukey’s *post hoc* test was used. For data with multiple time points and groups (e.g., cell proliferation assays), two-way analysis of variance followed by Tukey’s *post hoc* test was used. All statistical analyses were performed using GraphPad Prism software (version 9.0, GraphPad Software, San Diego, CA, USA, www.graphpad.com). A *P*-value < 0.05 was considered statistically significant. All bioinformatics analyses in this study were performed using the R programming language (version 4.2, https://www.r-project.org/) with a significance level set at *P* < 0.05. Pearson correlation analysis was employed to investigate the correlation between continuous variables.

## Results

### Quality control of single-cell sequencing analysis

**Figure 1:**
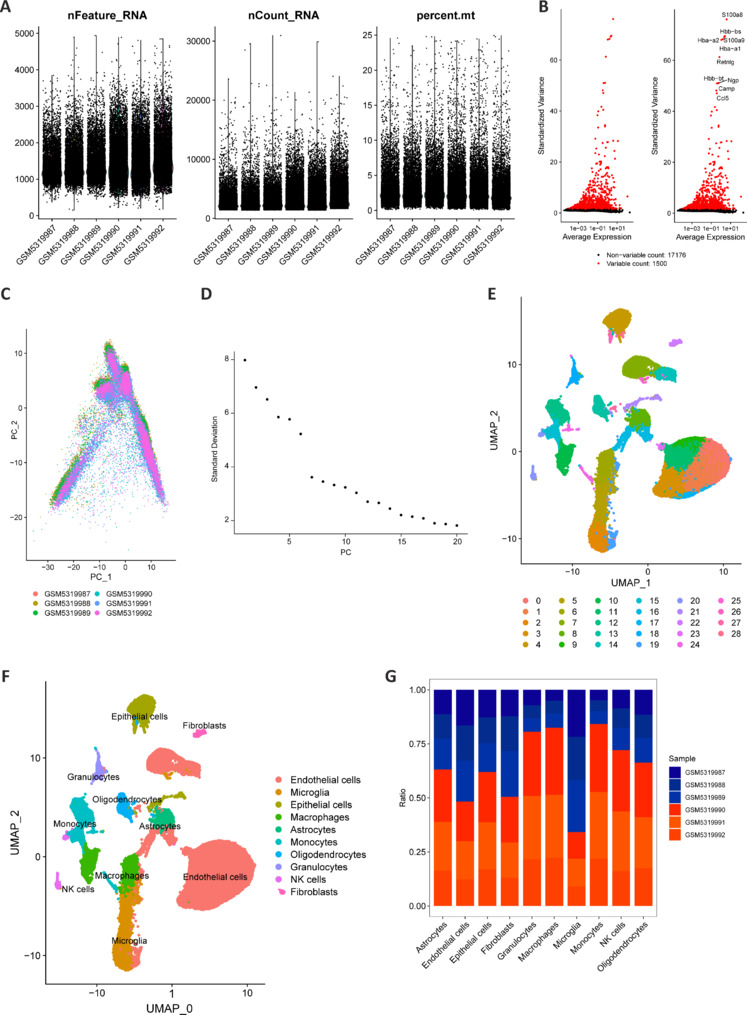
Marked increase in macrophage and monocyte cell counts after MCAO. (A) Number of unique genes detected (nFeature_RNA), percentage of mitochondrial genes (percent.mt), and total number of detected molecules (nCount_RNA) after quality control in the GSE174574 dataset. (B) Variance plot showing the top-10 most variable genes. (C) No prominent batch effects were detected by PCA. (D) Elbow plot to identify the optimal number of principal components. (E) UMAP visualization showing different cell clusters captured from scRNA-seq data. (F) In total, 29 cell clusters were annotated into 10 different cell types. (G) Bar plot showing the cell proportion in each sample by cell type. MCAO: Middle cerebral artery occlusion; PCA: principal component analysis; UMAP: Uniform Manifold Approximation and Projection.

The GSE174574 dataset comprises six individual samples. Preliminary sample screening was conducted based on the criteria of nFeature_RNA > 50 and percent.mt < 25 (**Figure 1A**). The top-10 genes with the highest standard deviation are shown (**Figure 1B**). We used PCA for dimensionality reduction and observed no prominent batch effects between samples (**Figure 1C**). Using the elbow plot method, we determined the optimal number of principal components to be 17 (**Figure 1D**). Subsequently, we performed cell clustering based on the similarity of gene expression between cells, ultimately yielding 29 distinct subgroups by UMAP analysis (**Figure 1E**).

### Cellular heterogeneity and increased monocyte abundance in ischemic stroke

In this study, 29 clusters were annotated into 10 distinct cell categories: endothelial cells, microglia, epithelial cells, macrophages, astrocytes, monocytes, oligodendrocytes, granulocytes, natural killer (NK) cells, and fibroblasts (**Figure 1F**). Notably, monocytes showed a relative increase in cell content in stroke samples (**Figure 1G**), which could be associated with inflammatory responses and blood–brain barrier disruption. Stroke is an acute cerebrovascular disease that disrupts blood supply to certain brain tissues, triggering inflammatory responses. Macrophages and monocytes are crucial cell types in the immune system and play a pivotal role during inflammation. When stroke occurs, macrophages and monocytes are recruited to damaged brain tissue to clear cellular debris, regulate immune responses, and promote tissue repair.

Subsequently, to comprehensively characterize the temporal evolution features of monocytes after ischemic stroke, we generated a temporal atlas of the major cell types in the ischemic hemisphere at single-cell resolution (**Additional Figure 1A–C**). Five time points (4 hours, 1 day, 2 days, 3 days, and 7 days post-MCAO) were selected to represent transcriptomic profiling across hyperacute and acute phases of cerebral ischemia. Consistent with observations in **Figure 1G**, a relative increase of monocyte content was identified in the ischemic hemisphere compared with the control group (**Additional Figure 1D**). Furthermore, the proportion of monocytes in the ischemic hemisphere at 1 day, 2 days, and 3 days post-MCAO was higher compared with the control group, gradually increasing from 1 day to 2 days, peaking at 2 days, and then declining (**Additional Figure 1E**). This highlights the proactive response of monocytes to the ischemic event in the acute phase of stroke and reveals dynamic changes in monocytes post-ischemic stroke.

### Intercellular communication network analysis shows monocyte-mediated crosstalk in the ischemic brain

**Figure 2:**
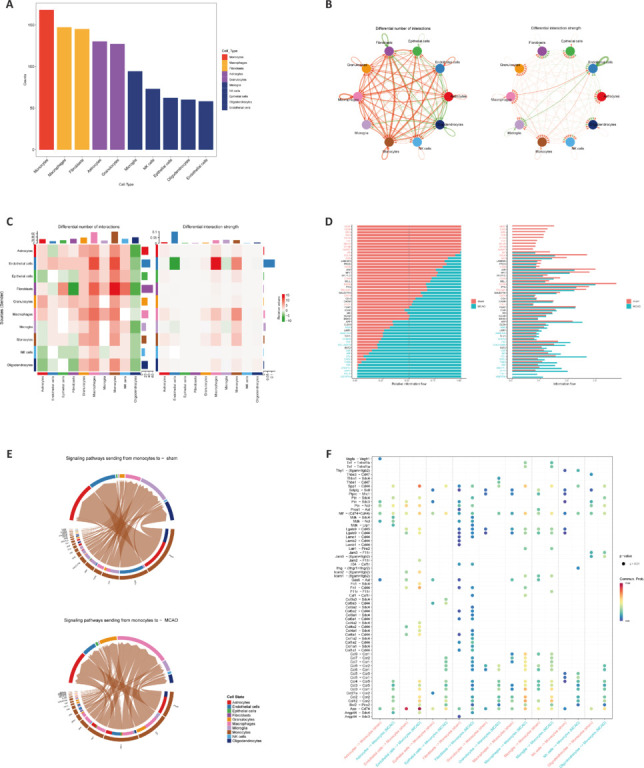
Analysis of cellular communication networks. (A) Bar graph showing the interaction count of each cell population. (B) Circle plot visualizing the differential number and strength of cell–cell communication interactions between sham and MCAO samples. Red (or green) edges indicate an increase (or decrease) in signal transmission in the MCAO group compared with the sham group. (C) Heatmap showing differential number and strength of interactions among different cell populations in the sham group and MCAO group. Color bar indicating that red (or green) represents increased (or decreased) signaling in the MCAO group compared with the sham group. (D) Bar plot comparing relative information flow between sham and MCAO conditions. The top signaling pathways enriched in the sham group are colored red; those colored green are enriched in the MCAO group. (E) Chord diagram for visualizing cell–cell communication between different cell groups. (F) Bubble plot showing significant ligand–receptor interactions between monocytes and other cells. MCAO: Middle cerebral artery occlusion.

We conducted ligand–receptor relationship analysis of features in the single-cell expression profiles using “CellChat” software. Notably, we found a closer potential interaction of monocytes with other cell types (**Figure 2A**). Subsequently, we observed complex interaction patterns among these cell subtypes, with significant communication differences between the sham and MCAO groups (**Figure 2B**). We found a greater amount of intercellular communication in MCAO samples compared with sham samples. The heatmap displayed a significant increase in both the number and strength of interaction when monocytes were targeted, compared with the control group, with the highest increase in interaction number (**Figure 2C**). Consequently, we focused on the interaction mechanisms between monocytes and other cell subgroups. We observed an increased interaction number between monocytes and fibroblasts, endothelial cells, and macrophages compared with the sham group, with enhanced interaction strength between monocytes and endothelial cells as well as macrophages (**Figure 2C**). To gain detailed understanding of the activated communication pathways post-MCAO, we compared the relative information flow between the two groups. Stacked bar graphs revealed major enriched activation pathways in MCAO samples including visfatin, interferon-II, osteopontin (SPP1), angiopoietin-like protein, and tumor necrosis factor (TNF) (**Figure 2D**). Significantly active pathways from monocytes to other cell types included the thrombospondin (THBS) and SPP1 signaling pathways in MCAO samples (**Figure 2E**). In the THBS pathway, monocytes interacted with the remaining nine cell subtypes, while in the SPP1 pathway, they only interacted with granulocytes and macrophages. Subsequently, we analyzed communication probabilities between cell clusters mediated by ligand–receptor pairs (**Figure 2F**). Compared with the sham group, the Spp1–Cd44 pair in MCAO samples exhibited increased signaling between monocytes and other cells. This indicates higher communication strength, especially in the interaction between endothelial cells and monocytes. MCAO treatment promoted enhanced communication pathways from fibroblasts and macrophages to monocytes, primarily focused on chemokine (C-C motif) ligand (CCL) signals.

Subsequently, we investigated communication relationships between monocytes and other cell clusters in the MCAO-specific signaling pathways, SPP1 and TNF. MCAO treatment enhanced the signal strength of the SPP1 and TNF pathways in monocytes, with the strongest SPP1 communication observed when endothelial cells acted as senders and monocytes as targets (**Additional Figure 2A–D**). Through network centrality analysis of SPP1 and TNF, we identified monocytes and macrophages as crucial cells regulating the SPP1 signaling network, with monocytes being the primary signal receivers (**Additional Figure 2E**). In the TNF communication network, monocytes primarily played roles as mediators and influencers (**Additional Figure 2F**). Furthermore, contribution analysis of regulatory factors in these pathways emphasized the significant contributions of Spp1–Cd44 in the SPP1 signaling pathway and Tnf–Tnfrsf1a in the TNF pathway (**Additional Figure 2G** and **H**). **Additional Figure 2I** illustrates the communication interactions between other cell clusters and monocytes in the SPP1 and TNF pathways.

### Development of a robust monocyte-based gene signature for stroke diagnosis

**Figure 3:**
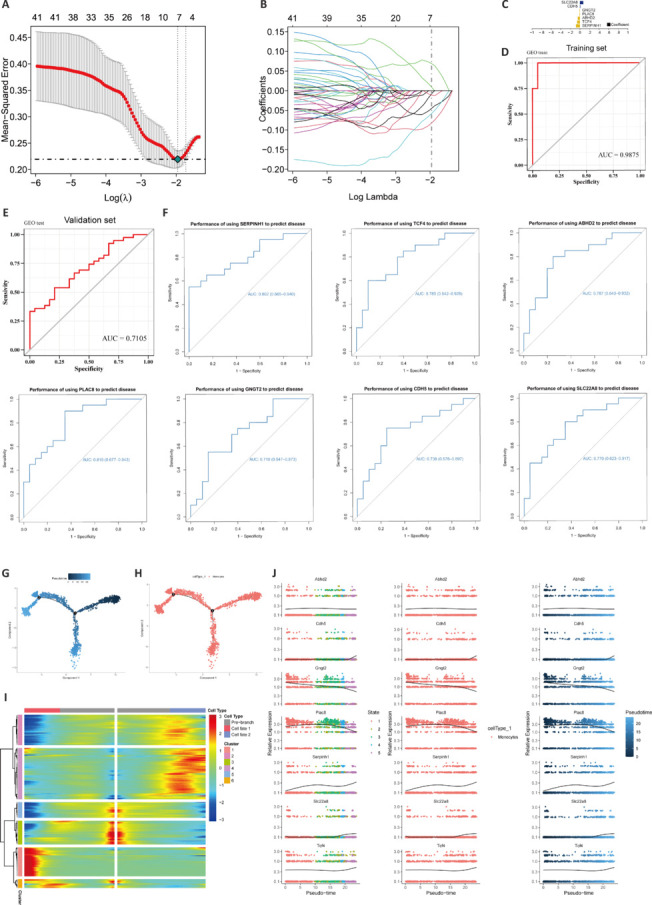
Construction and performance validation of a prediction model based on monocyte gene expression. (A) Cross-validation curve of Lasso regression analysis. (B) Distribution plot of Lasso coefficients for variable selection. (C) Bar plot showing Lasso regression coefficients for key genes related to ischemic stroke. (D, E) Performance evaluation of the predictive model by ROC curves in the training set (D) and validation set (E). (F) Performance evaluation of key genes by ROC curves. (G) Cell trajectory skeleton diagram. (H) Pseudotime trajectory chart. (I) Analysis heatmap of branching points. (J) Expression changes of key genes with pseudotime values, cell type, and state. LASSO: Least Absolute Shrinkage and Selection Operator; ROC: receiver operating characteristic.

We used the GSE22255 dataset as our training set and GSE16561 dataset as our validation set. A total of 331 monocytes genes were selected for feature selection using Lasso regression. Lasso regression identified seven genes as characteristic genes for ischemic stroke (**Figure 3A–C**). The predictive model constructed using these seven genes showed strong diagnostic performance, with an AUC of 0.9875 (**Figure 3D**). Subsequently, we used the GSE16561 dataset as an external dataset to further validate the diagnostic model. This validated the model’s predictive value, with an AUC of 0.7105 (**Figure 3E**). Using the expanded validation datasets (GSE16561, GSE37587, and GSE124026), the model exhibited a prominent and robust predictive performance with an AUC of 0.7112 (**Additional Figure 2J**). For diagnostic performance validation, the predictive efficacy of these seven key genes for ischemic stroke was investigated by receiver operating characteristic curve analysis. Higher AUC values indicate better predictive performance, as shown in **Figure 3F**. Altogether, these results suggest that all seven key genes could effectively predict the onset and progression of disease.

### Monocyte development trajectory and dynamic expression patterns of key genes

To investigate the developmental dynamics and potential cell fate transitions of monocytes in the context of ischemic stroke, we performed single-cell trajectory analysis using the Monocle algorithm. This approach computationally orders cells along a pseudotime trajectory based on their transcriptional similarity, enabling the inference of developmental trajectories and critical transition points.

The pseudotime trajectory revealed a striking bifurcation structure (**Figure 3G** and **H**), indicating the existence of two distinct developmental paths or cellular states of monocytes. We visualized the cells in two-dimensional space using the first two principal components and colored them according to their pseudotime values (**Figure 3G**) or assigned cell types (**Figure 3H**). All monocytes originated from a common starting position at the beginning of the trajectory (pseudotime 0) and gradually diverged, ultimately forming two separate branches. This bifurcation suggests that monocytes may undergo different fate decisions or functional specialization during the course of ischemic stroke.

Differential gene expression analysis at the branching point identified key transcriptional changes associated with monocyte fate determination and functional specialization (**Figure 3I**). The differentially expressed genes clustered into six distinct modules, representing specific molecular pathways and regulatory mechanisms. These branch-specific gene signatures provide valuable insights for further investigation of monocyte functional subsets and gene regulatory programs in the context of ischemic stroke.

We specifically examined the dynamic expression changes of our seven characteristic key genes (ABHD2, CDH5, GNGT2, PLAC8, SERPINH1, SLC22A8, and TCF4) along the pseudotime axis (**Figure 3J**). Notably, PLAC8 and GNGT2 showed high expression levels in early pseudotime stages, followed by a gradual decline along one of the branches. This expression pattern suggests that PLAC8 and GNGT2 may play important roles in the initial response of monocytes to ischemic injury or the maintenance of a more immature state. In contrast, SERPINH1 and TCF4 showed increased expression towards later stages of pseudotime, particularly along one branch, indicating their potential involvement in differentiation or functional maturation of a specific monocyte subset.

### Association of key genes with immune cell infiltration and immunomodulatory landscape

**Figure 4:**
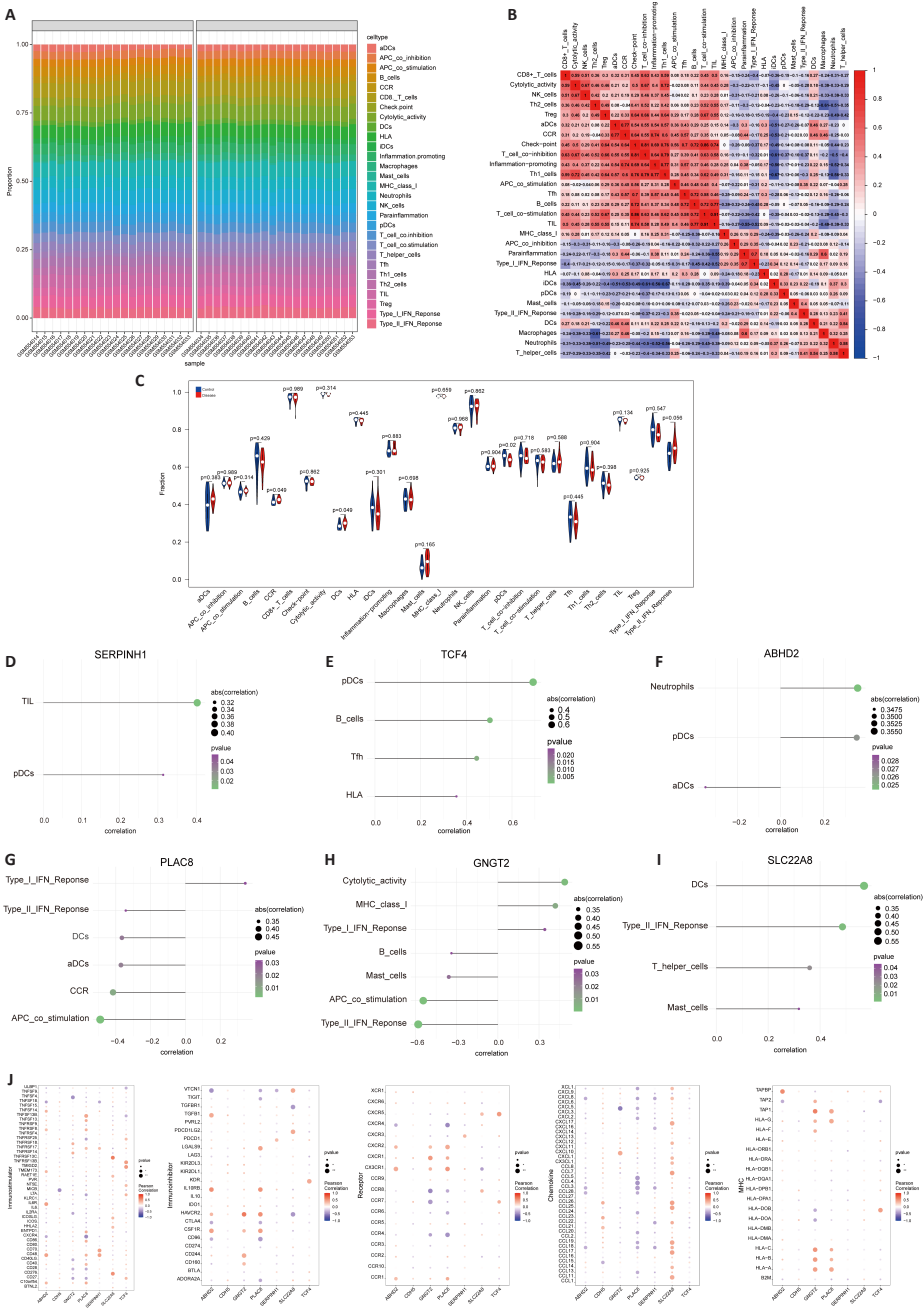
Immune infiltration landscape after ischemic stroke. (A) Immunological components and relative abundance in samples. (B) Heatmap showing correlation between immune cell types. (C) Violin plot showing relative abundance of immune cells in the control group and stroke patients. (D–I) Interaction between expression levels of key genes and immune cell abundance in ischemic stroke. (J) Bubble charts showing the interplay of key genes with immunostimulators, immunoinhibitors, cell receptors, chemokines, and major histocompatibility complex (MHC). *P < 0.05, **P < 0.01, ***P < 0.001.

The immune microenvironment significantly impacts diagnosis, survival outcomes, and clinical treatment sensitivity of major diseases. By analyzing the relationship between key genes in disease datasets and immune infiltration, we aimed to explore the mechanisms through which these key genes affect the progression of ischemic stroke. The immune cell content of each patient is illustrated in **Figure 4A**. Multiple significant correlation pairs are observed among immune infiltration levels (**Figure 4B**). Moreover, when compared with normal patients, samples from the disease group had significantly higher levels of chemokine receptors (CCR) and dendritic cells (DCs) (**Figure 4C**). Elevation of CCR may indicate increased receptor expression of immune cells in stroke samples, thereby enhancing their responsiveness to chemokines. This could lead to increased immune cell infiltration around the stroke lesion, regulating inflammatory responses, and promoting tissue repair. The significant increase in DC levels may suggest activation and increased infiltration of DCs in stroke samples, contributing to inflammatory responses and immune regulation processes. We further explored the relationship between key genes and immune cells, discovering several key genes highly correlated with immune cells. SERPINH1 was significantly positively correlated with tumor-infiltrating lymphocytes and pDCs (**Figure 4D**); TCF4 was significantly positively correlated with pDCs and B_cells (**Figure 4E**); ABHD2 was significantly positively correlated with Neutrophils and pDCs, and significantly negatively correlated with aDCs (**Figure 4F**); PLAC8 was significantly positively correlated with Type_I_IFN_Response and significantly negatively correlated with APC_co_stimulation and CCR (**Figure 4G**); GNGT2 was significantly positively correlated with Cytolytic_activity and MHC_class_I, and significantly negatively correlated with Type_II_IFN_Response and APC_co_stimulation (**Figure 4H**); and SLC22A8 was significantly positively correlated with DCs and Type_II_IFN_Response (**Figure 4I**). We further examined correlations between these key genes and various immune factors, including immune modulators, chemokines, and cell receptors, from the TISIDB database (**Figure 4J**). These analyses confirmed close association of these key genes with immune cell infiltration levels and their significant roles in the immune microenvironment.

### Critical pathways and molecular mechanisms regulated by key genes

**Figure 5:**
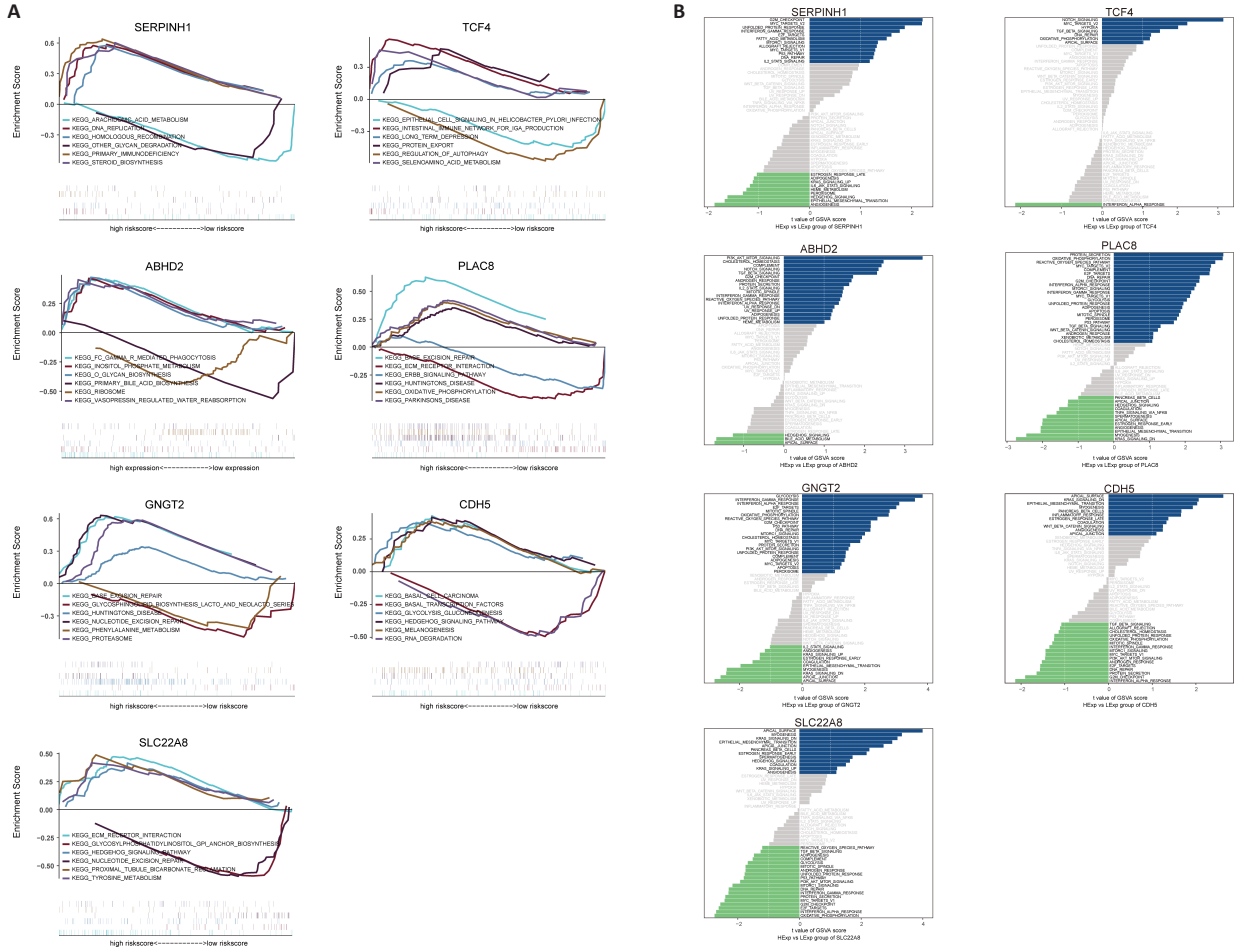
GSEA and GSVA analyses to identify principal enrichment patterns of key genes in ischemic stroke. (A) GSEA analysis of key genes. (B) GSVA analysis of key genes. GSEA: Gene set enrichment analysis; GSVA: gene set variation analysis.

In our subsequent analysis, we wanted to examine the specific signaling pathways that show enrichment by key genes, with the objective of understanding the potential molecular mechanisms through which these key genes exert their influence on the progression of ischemic stroke. The results of our GSEA analysis identified distinct enrichment patterns for these key genes (**Figure 5A**). High expression of SERPINH1 was significantly associated with the enrichment of pathways such as DNA replication, homologous recombination, primary immunodeficiency, and steroid biosynthesis. Increased expression levels of TCF4 showed significant enrichment in pathways including intestinal immune network for IgA production, long term depression, protein export, and selenoamino acid metabolism. ABHD2 showed a pronounced enrichment pattern in pathways related to FC gamma R-mediated phagocytosis, inositol phosphate metabolism, O glycan biosynthesis, and vasopressin regulated water reabsorption. PLAC8 showed predominant enrichment in pathways encompassing base excision repair, Huntington’s disease, oxidative phosphorylation, and Parkinson’s disease. High expression of GNGT2 was significantly associated with the enrichment of pathways including base excision repair, Huntington’s disease, nucleotide excision repair, and proteasome. CDH5 had a prominent enrichment pattern in pathways such as basal cell carcinoma, glycolysis gluconeogenesis, hedgehog signaling pathway, and melanogenesis. SLC22A8 showed principal enrichment in pathways related to extracellular matrix receptor interaction, hedgehog signaling pathway, proximal tubule bicarbonate reclamation, and tyrosine metabolism.

### Gene set variation analysis identifies key gene involvement in stroke-related signaling pathways

The results of GSVA identified specific signaling pathways enriched by individual key genes, providing insight into their potential roles in the onset and progression of stroke (**Figure 5B**). Notably, SERPINH1 showed enrichment in pathways associated with G2/M checkpoint, Myc targets V2, and unfolded protein response. TCF4 was primarily enriched in pathways related to Notch signaling, Myc targets V2, and hypoxia. ABHD2 showed significant enrichment in pathways such as PI3K–AKT–mTOR signaling, cholesterol homeostasis, and complement. PLAC8 was enriched in pathways encompassing protein secretion, oxidative phosphorylation, and ROS pathway. GNGT2 showed enrichment in pathways including glycolysis, interferon gamma response, and interferon alpha response. CDH5 showed significant enrichment in pathways such as apical surface, Kras signaling DN, and epithelial mesenchymal transition. Lastly, SLC22A8 was found to be enriched in pathways related to apical surface, myogenesis, and Kras signaling DN. These findings collectively suggest that these pathways may exert an influence on the occurrence and development of stroke.

### miRNA–mRNA regulatory networks, transcriptional regulation analysis, and potential drug interactions of key genes

**Figure 6:**
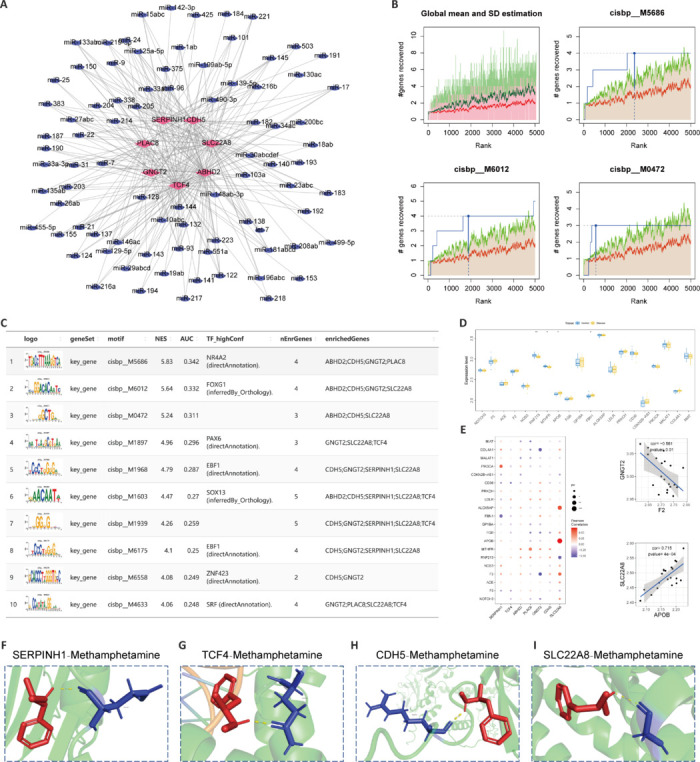
Regulatory mechanisms of key genes and their correlation with ischemic stroke–regulating genes. (A) miRNA regulatory network of key genes. (B) Cumulative recovery curves of transcription factor enrichment analysis. (C) Table of enriched motif annotation analysis with top-10 ranked normalized enrichment score (NES) scores. (D) Boxplot showing expression analysis of ischemic stroke-related disease genes with top-20 correlation scores. (E) Interactions between expression levels of seven key genes and ischemic stroke–related genes. (F–I) Molecular docking images showing interaction of SERPINH1 (F), TCF4 (G), CDH5 (H), and SLC22A8 (I) with methamphetamine. *P < 0.05, **P < 0.01, ***P < 0.001. miRNA: MicroRNA; NES: normalized enrichment score.

Next, we conducted a reverse prediction using our seven key genes in the mircode database. This resulted in a total of 83 miRNAs and 295 mRNA–miRNA interaction pairs (**Figure 6A**). The set of key genes identified was subject to regulation by multiple common mechanisms, including several transcription factors. Enrichment analysis of these transcription factors was performed using cumulative recovery curves (**Figure 6B**), revealing significant enrichment of the cisbp__M5686 motif for four key genes, with a calculated NES of 5.83. The enriched motifs and corresponding transcription factors for key genes (**Figure 6C**) indicated nuclear receptor 4A2 (NR4A2) as the primary transcription factor for ABHD2, CDH5, GNGT2, and PLAC8. NR4A2 plays a critical regulatory role in neural development, cell proliferation, and maturation. It is closely linked to neurological disorders, regulating pro-inflammatory mediators, and displaying neuroprotective effects by modulating various signals (Jakaria et al., 2019). Furthermore, NR4A2 acts as a downstream target in recombinant tissue-type plasminogen activator therapy, being linked to endothelial dysfunction and complications associated with this treatment during the ischemic stroke period (Merino-Zamorano et al., 2015). Potential drugs interactions of these key genes were predicted using the CTD database. Molecular docking was conducted using AutoDock software. The proteins and compounds selected for the key genes were: SERPINH1: P50454-Methamphetamine; TCF4: 6OD3-Methamphetamine; CDH5: P33151-Methamphetamine; and SLC22A8: Q8TCC7-Methamphetamine. Docking poses with the lowest binding energies are shown in **Figure 6F–I**. Molecular docking revealed binding energies of –4.14 kcal/mol for the interaction between SERPINH1 and Methamphetamine, –3.90 kcal/mol for TCF4 and Methamphetamine, –4.58 kcal/mol for CDH5 and Methamphetamine, and –4.51 kcal/mol for SLC22A8 and Methamphetamine (**Figure 6F–I**). Additionally, all four key genes exhibited hydrogen bonding interactions with Methamphetamine.

### Correlations of key genes with established ischemic stroke–related genes

We obtained ischemic stroke-related disease genes from the GeneCards database (https://www.genecards.org/). Analyzing inter-group expression differences among disease genes, we identified differential expression of the *RNF213*, *MTHFR*, *APOB*, and *FBN1* genes in two patient groups (**Figure 6D**). Subsequently, we analyzed expression levels of the seven key genes and top-20 genes by relevance score. This found significant correlations between expression levels of these seven genes and multiple disease-related genes. Notably, GNGT2 showed significant negative correlation with F2 (cor = –0.561), while SLC22A8 showed significant positive correlation with APOB (cor = 0.715) (**Figure 6E**).

### Cellular distribution and immune-related co-expression patterns of key genes

**Figure 7:**
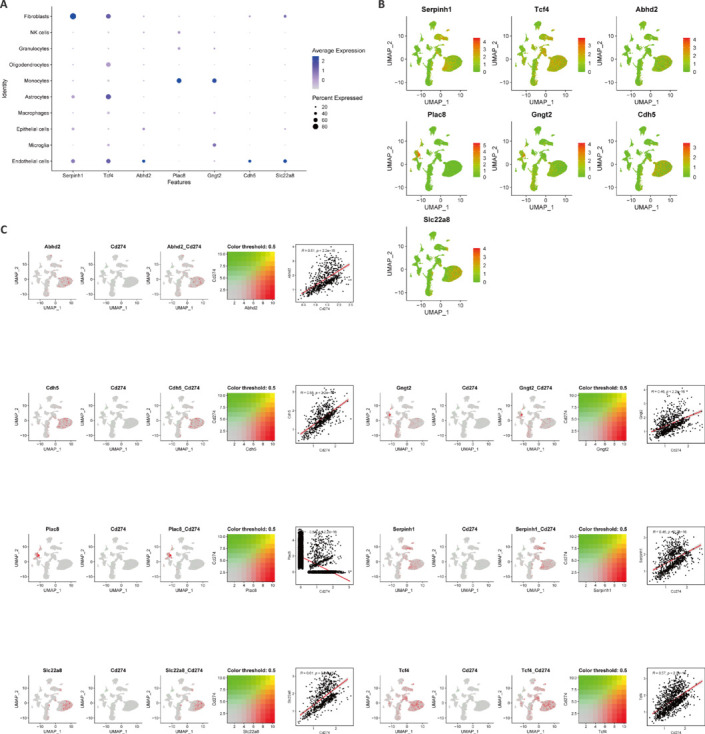
Cellular distribution of key genes and co-expression analysis with CD274 in GSE174574. (A) Bubble plot showing expression levels of key genes. (B) UMAP visualization of the distribution and expression of key genes across 10 cell types. (C) Co-expression analysis of key genes with the immune-related gene, CD274. UMAP: Uniform manifold approximation and projection.

**Figure 8:**
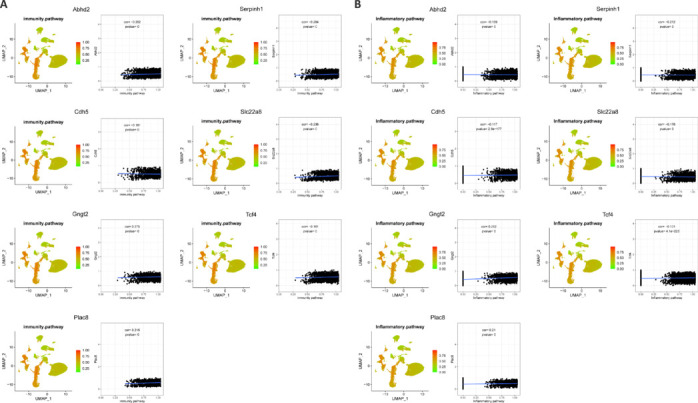
Correlation analysis of key genes with immune inflammatory pathways. (A) Interactions between key genes and immune pathways. (B) Correlation analysis of key genes with inflammatory pathways.

Expression of the seven key genes in 10 different cell types is illustrated in **Figure 7A** and **B**. CD274 plays a significant role in immune regulation by inhibiting activated immune cells via binding to PD-1, thereby mitigating the extent of inflammatory reactions. Therefore, CD274 may be involved in inflammation regulation during the course of a stroke. Subsequently, we visualized co-expression of these key genes with the immune-related gene, CD274, across 10 cell markers (**Figure 7C**) to provide further insight into immune mechanisms. ABHD2, CDH5, SLC22A8, GNGT2, SERPINH1, and TCF4 showed positive correlations with expression of CD274, while PLAC8 exhibited negative correlation. SERPINH1, TCF4, and GNGT2 are expressed in multiple cell subtypes. PLAC8 and GNGT2 show highest expression levels in monocytes, comprising the highest proportion. This provides potential targets for the development of immune modulation therapies. Furthermore, we conducted correlation analysis between the key genes and inflammatory pathways as well as immune pathways using single-sample GSEA (**Figure 8**). PLAC8 and GNGT2 were positively correlated with both inflammatory and immune pathways, while the remaining key genes were negatively correlated with these pathways.

### Validation of differential expression of key genes in peripheral monocytes post-stroke

**Figure 9:**
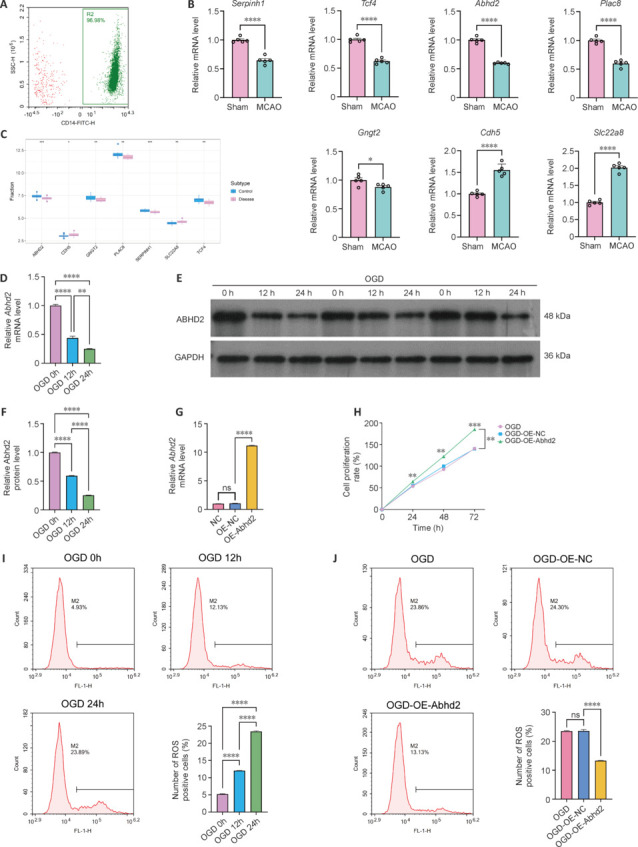
Validation of key genes and investigation of Abhd2 function post-stroke. (A) Flow cytometry assessment of CD14^+^ monocytes. (B) Quantification of expression of key genes in peripheral blood monocytes post-MCAO by qRT-PCR (*n* = 5). Data are expressed as mean ± SEM and were analyzed by Student’s *t*-test. (C) Boxplot showing expression analysis of key genes between the control group and stroke group from the transcriptome data. Analysis of Abhd2 expression changes in monocytes at OGD-0h, -12h, and -24h by qRT-PCR (D) and western blot analysis (E, F) (*n* = 3). (G) Quantification of *Abhd2* mRNA expression by qRT-PCR in monocytes from NC, OE-NC, and OE-Abhd2 groups (*n* = 3). (H) Measurement of monocyte proliferation post-OGD at days 1, 2, and 3 using the MTS assay (*n* = 3). Data are expressed as mean ± SEM and were analyzed by two-way analysis of variance with Tukey’s *post hoc* test. (I) Flow cytometry evaluation of ROS expression in monocytes at OGD-0h, -12h, and -24h (*n* = 3). (J) Flow cytometry analysis of ROS expression in monocytes from NC, OE-NC, and OE-Abhd2 groups (*n* = 3). *P < 0.05, **P < 0.01, ***P < 0.001, ****P < 0.0001. In D, F, G, I, and J, data are expressed as mean ± SEM and were analyzed by one-way analysis of variance with Tukey’s *post hoc* test. MCAO: Middle cerebral artery occlusion; MTS: 3-(4,5-dimethylthiazol-2-yl)-5-(3-carboxymethoxyphenyl)-2-(4-sulfophenyl)-2H-tetrazolium; NC: negative control; OE: overexpression; OGD: oxygen–glucose deprivation; qRT-PCR: quantitative reverse transcription-polymerase chain reaction; ROS: reactive oxygen species; SEM: standard error of the mean.

Building upon our previous analyses, we next isolated monocytes from peripheral blood of experimental mice (**Figure 9A**) and examined the expression of our seven key genes within these monocytes using qRT-PCR (**Figure 9B**). Compared with the control group, expression levels of *Serpinh1*, *Tcf4*, *Abhd2*, and *Plac8* were significantly reduced in MCAO mice. *Gngt2* also displayed a slight decreasing trend post-MCAO. Conversely, *Cdh5* and *Slc22a8* showed higher expression levels than in the control group. The expression profile changes of key genes in peripheral blood mononuclear cells (**Figure 9C**) exhibited a similar trend.

### The regulatory role of Abhd2 in monocyte proliferation and oxidative stress response

We further investigated the role of Abhd2 in monocytes. Here, we examined dynamic changes of Abhd2 within monocytes and its correlation with ischemic injury. qRT-PCR showed a significant decrease in Abhd2 expression at OGD-12h, with a further decline after 24 hours of OGD (**Figure 9D**). Western blotting showed a similar trend (**Figure 9E** and **F**). To further substantiate the role of Abhd2, *in vitro* experiments were conducted to explore the effect of Abhd2 on the biological function of monocytes. Monocytes were transfected with OE-Abhd2 plasmids in an OGD model to induce Abhd2 overexpression (**Figure 9G**). Using the MTS assay, primary cultured monocytes showed a significant increase in proliferation at just 1 day after transfection with the OE-Abhd2 plasmid (*P* < 0.01; **Figure 9H**); this effect was sustained for at least three days (*P* < 0.001; **Figure 9H**). Additionally, ROS production in monocytes under ischemic conditions was examined. Flow cytometry analysis showed a notable increase in the proportion of ROS^+^ monocytes at OGD-12h and OGD-24h (**Figure 9I**). Moreover, as the treatment duration extended, the number of ROS-producing monocytes increased. Notably, at OGD-24h, there was a significant enhancement in ROS-producing monocytes compared with OGD-12h (*P* < 0.0001; **Figure 9I**). Excessive production of ROS induces oxidative stress responses, a critical mechanism of cell damage following ischemia (Shirley et al., 2014). Remarkably, Abhd2 overexpression significantly reduced the ROS^+^ monocyte proportion (*P* < 0.0001; **Figure 9J**).

### Enhancement of phagocytic activity in monocytes via Abhd2 overexpression

**Figure 10:**
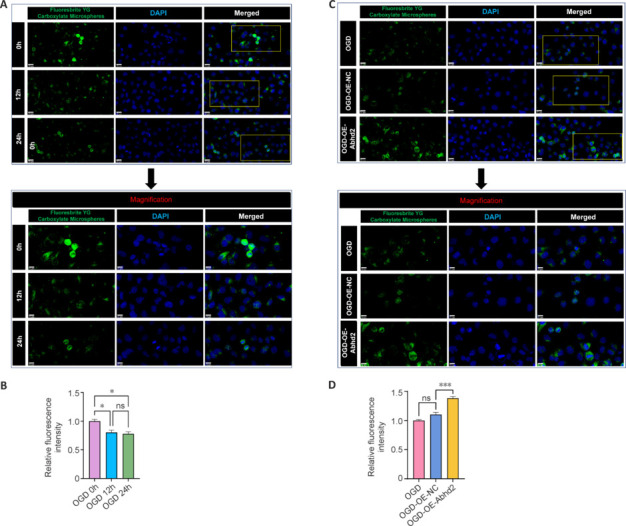
The effect of Abhd2 overexpression on phagocytic activity of monocytes. (A) Representative images showing the uptake of Fluoresbrite YG carboxylate microspheres by monocytes at OGD-0h, -12h, and -24h (scale bar: 20 μm). (B) Quantification of monocytes engulfing fluorescently labeled beads at OGD-0h, -12h, and -24h (*n* = 3). (C) Representative images showing the uptake of Fluoresbrite YG carboxylate microspheres by monocytes in the OGD, OGD-OE-NC, and OGE-OE-Abhd2 groups (scale bar: 20 μm). (D) Quantification of monocytes engulfing fluorescently labeled beads in the OGD, OGD-OE-NC, and OGD-OE-Abhd2 groups (*n* = 3). OE-Abhd2: Abhd2 overexpression plasmid; OE-NC: empty vector; OGD: oxygen–glucose deprivation.

Consequently, we investigated the impact of Abhd2 on phagocytic activity of monocytes. The phagocytic function of monocytes is pivotal for both damage and repair post-stroke. Immunofluorescence phagocytosis assays confirmed a significant reduction in monocyte phagocytic activity under OGD-12h and OGD-24h treatments (**Figure 10A** and **B**). Compared with the OGD-NC group, the Abhd2 overexpression group exhibited a higher uptake of fluorescent microspheres by monocytes, significantly enhancing their phagocytic activity (*P* < 0.001; **Figure 10C** and **D**).

## Discussion

After a stroke, perivascular immune cells can directly impact the integrity of the blood–brain barrier. Blood-derived monocytes/macrophages, neutrophils, and T lymphocytes also infiltrate ischemic brain tissue, increasing blood–brain barrier permeability and secreting inflammatory mediators. In contrast, these cells promote blood–brain barrier repair and angiogenesis in the later stages of ischemic stroke. Single-cell sequencing in the brain tissue of mice at 24 hours after MCAO found that monocytes showed the most significant change in cellular abundance. Compared with the control group, there was a relative increase in monocyte number during the acute phase of ischemic stroke, highlighting the heterogeneity of monocytes and their rapid response to cerebral ischemia. This is consistent with the literature, whereby Ritzel et al. observed a notable increase in monocyte quantity within the brains of MCAO mice at 70 hours after surgery. They showed that monocytes played a primary phagocytic role and actively participated in the early clearance of cellular debris (Ritzel et al., 2015). CCR2Ly6C^++hi^ monocytes are presumed to be “inflammatory” (Chu et al., 2014). At 24 hours after stroke, Ly6C^hi^ monocytes increased in peripheral blood and brain tissue, accompanied by a decrease in Ly6C^lo^ monocytes. Treatment with INCB3344 reversed this trend but exacerbated post-stroke functional deficits and increased the infarct area (Chu et al., 2015). This suggests that an increase of Ly6C^hi^ monocytes in brain tissue plays a protective role in acute cerebral ischemia. In addition, based on the frequency and intensity of intercellular communication, monocytes exhibited stronger intercellular communication capabilities. Once they reach brain tissue, monocytes not only exert their own influence but also coordinate the functionality of brain cells. The active communication and immunoregulatory role of these cells may potentially contribute to modulation of disease progression. For example, monocytes can produce platelet-derived growth factors, inducing mitotic activity in endothelial cells (Jaipersad et al., 2014). Additionally, CCR2 monocytes can regulate the proliferation and angiogenic properties of astrocytes post-brain vascular injury via interleukin (IL)-6 secretion, collectively promoting functional recovery after ischemic stroke (Pedragosa et al., 2020; Choi et al., 2023). Our analysis builds upon this previous research by bioinformatic demonstration of active communication between monocytes and various cell types. Our findings emphasize their key role in the pathophysiology of ischemic stroke and their significance in disease research.

The intricate network formed by interactions between circulating monocytes and infiltrating monocytes has profound effects on stroke. Subsequent to a stroke, classical monocytes undergo significant expansion in the brain and blood, which restricts the progression of ischemic infarction, alleviates functional impairments, and promotes the polarization of M2 macrophages (Chu et al., 2015). Simultaneously, immature monocytes recruited from the periphery to ischemic brain tissue gradually acquire the expression of typical macrophage markers, demonstrating potential to differentiate into macrophages (Miró-Mur et al., 2016). A prospective study also indicated association between increased peripheral monocyte counts and heightened stroke risk (Zhong et al., 2023). Furthermore, a study on remote limb ischemic preconditioning therapy, where the transition of circulating monocytes in stroke mice were promoted towards a pro-inflammatory phenotype, led to reduced brain injury and improved gait function in chronic stroke (Yang et al., 2019). The significance of peripheral monocytes in the development and treatment of stroke cannot be overlooked.

Through bulk transcriptome analysis and experiments on mice using the MCAO model, we found close association of seven key genes (*SERPINH1*, *TCF4*, *ABHD2*, *PLAC8*, *GNGT2*, *CDH5*, and *SLC22A8*) with the development of ischemic stroke. Consistent with our validation of key genes, existing studies have found differential expression of SERPINH1 in blood samples of ischemic stroke patients compared with controls, with downregulation in the stroke group (Zhu et al., 2020; Cheng et al., 2024). Understanding the role of monocytes in stroke is growing. Monocytes and their synthesized and metabolic products are crucial factors in regulating inflammation and thrombosis. Monocyte-platelet aggregates serve as significant markers of platelet activation during the acute phase of ischemic stroke, reflecting pre-thrombotic and inflammatory processes following stroke and potentially correlating with immune responses (McCabe et al., 2004; Htun et al., 2006; Ishikawa et al., 2012). Monocyte infiltration is also a critical aspect of brain injury repair. On the 2^nd^ and 3^rd^ days post-photothrombotic stroke surgery, significant infiltration of monocytes is observed in structurally intact infarct areas (Werner et al., 2020). SERPINH1 plays a role in promoting thrombus formation (Sasikumar et al., 2018), and its inhibitor can inhibit platelet aggregation and alleviate cerebral ischemic injury in MCAO rats (Wu et al., 2021). Therefore, investigating the impact of SERPINH1 on stroke outcomes by regulating thrombotic burden and its direct role in brain ischemic injury is a worthwhile area of exploration. TCF-4 participates in the neuroprotective effects of oxymatrine and morroniside following cerebral ischemia–reperfusion injury as a component of the Wnt/β-catenin pathway (Sun et al., 2014; Lan et al., 2023). TCF4 is closely associated with the generation of DCs derived from monocytes and acts as a functional marker during the differentiation process of CD16^+^ monocytes into DCs (Wacleche et al., 2018). As a stroke-related key gene in monocytes, TCF4 is involved in both signaling pathways regulating stroke injury and as a functional marker of monocytes. These findings offer potential directions for investigating the evolution of monocyte roles following a stroke. PLAC8 potentially regulates autophagy and pro-inflammatory cytokine release in monocytes. In monocytes, PLAC8 demonstrates reactivity to inflammation and mediates the synthesis of inactive IL-1β and IL-18 precursors by regulating monocyte autophagy (Segawa et al., 2018; Zevallos et al., 2023). Existing studies on GNGT2 have mainly focused on light signal transduction pathways. Only one study identified an association between Gngt2 and atherosclerosis and monocytes, showing differential expression of monocyte Gngt2 under atherosclerotic conditions and enrichment of chemotactic signaling pathways (Sarad et al., 2023). Our study broadens the perspective on GNGT2 in monocytes and disease research. Vascular endothelial (VE)-cadherin, encoded by CDH5, is predominantly distributed at the junctions between endothelial cells, and mediates transendothelial migration of monocytes/macrophages (Hashimoto et al., 2011; Gerhardt and Ley, 2015; Koecke et al., 2024). While the Cdh5–Cre system is extensively used for selective gene targeting in endothelial cells and gene knockout promotion, its specific involvement in stroke remains inadequately researched. In stroke patients with cerebral infarction, serum levels of VE–cadherin are increased (He et al., 2017). This aligns with our experimental results showing significant increase of Cdh5 in peripheral blood monocytes of MCAO mice, providing a degree of mutual corroboration.

Although prior studies have not established the roles of peripheral SLC22A8, PLAC8, GNGT2, and CDH5 in ischemic stroke, their enrichment in pathways identified through GSEA or GSVA analyses suggests a potential regulatory role in stroke. SLC22A8 demonstrated enrichment in pathways associated with the hedgehog signaling pathway and tyrosine metabolism. Inhibition of protein tyrosine phosphatase can diminish hypoxic brain injury in preterm mice, fostering the recovery of damaged white matter (Wang et al., 2023), promoting neural repair following stroke, and expediting tissue restoration (Luo et al., 2022). PLAC8 exhibited enrichment in oxidative phosphorylation and ROS pathways. Amelteon, a melatonin receptor agonist, can attenuate oxidative stress-induced responses and inhibit neuronal autophagy, thereby reducing brain injury in the context of stroke (Zhang et al., 2024). Clearing ROS, alleviating oxidative stress-induced damage in mesenchymal stem cells, and enhancing cellular viability contributes to the improvement of ischemic stroke (You et al., 2023). Additionally, central post-stroke pain can be ameliorated by the inhibition of oxidative stress (Bai et al., 2024). Enrichment of the glycolysis pathway was observed with GNGT2. Alterations in cerebral glucose metabolism are closely associated with the primary pathogenic mechanism of ischemic stroke (Guo et al., 2023). Regulation of the glycolysis pathway can be achieved by inhibiting glycolytic enzymes and enabling inhibition of myeloid-derived suppressor cell differentiation through the blockade of mTOR signaling. This reduces the cerebral infarction size and alleviates brain damage in ischemic stroke (Yan et al., 2022). We have found significant enrichment patterns of CDH5 in the hedgehog signaling pathway. Regulatory involvement of the sonic hedgehog pathway in the pathophysiology of stroke has been established (Chen and Jin, 2023). Inhibition of the hedgehog signaling pathway can counteract the pro-angiogenic effects mediated by Dl-3-n-butylphthalide following stroke (Dai et al., 2023). Meanwhile, the smoothened-dependent sonic hedgehog pathway effectively improves brain damage and facilitates the recovery of neurological function in mice after MCAO by suppressing GLT-1 expression on cell membranes (Wang et al., 2021). Furthermore, disruption of the sonic hedgehog signaling pathway in stroke mice causes increased anxiety and delays the recovery process of motor function (Wang et al., 2022).

Within the key genes, Abhd2 emerged as a promising candidate for detailed investigation based on its predicted involvement in critical pathways. A consistent trend between *in vivo* and *in vitro* experimental outcomes underlines the robustness of our findings, providing compelling evidence for our bioinformatics predictions and emphasizing the rapid response of Abhd2 and monocytes to ischemic conditions. ABHD2 potentially influences the development of atherosclerosis and emphysema (Lv et al., 2024) by mediating the recruitment and infiltration of monocytes/macrophages. While there are no reported associations between ABHD2 and stroke, there is a close correlation between ABHD2 and atherosclerosis. Large artery atherosclerosis serves as a common source of embolism in the etiology of ischemic stroke (Campbell et al., 2019). ABHD2 exhibits high expression in macrophages within atherosclerotic plaques and is gradually upregulated during differentiation of peripheral blood monocytes into macrophages (Miyata et al., 2008), demonstrating stage-specific regulation. Consequently, ABHD2 may regulate the origin and development of stroke by modulating the recruitment/infiltration of monocytes. Following a stroke, bone marrow cells undergo rapid activation, with an increase in newly produced bone marrow-derived monocytes that are subsequently recruited to the brain (Denes et al., 2011; Ritzel et al., 2015). During single-cell analysis, an increased proportion of monocytes in post-stroke brain tissue was observed. Clearly, the proliferative status of monocytes is emerging as a pivotal element in the peripheral immune response to stroke. A regulatory role of ABHD2 in cell proliferation was shown in human epidermal melanocytes and human prostate cancer cells (Obinata et al., 2016; Zhao et al., 2021). In this study, Abhd2 also demonstrated regulatory capabilities over cell proliferation. Upregulation of Abhd2 expression was associated with a significant enhancement in monocyte proliferation capacity. Additionally, we investigated the impact of upregulated Abhd2 expression on monocyte ROS production. Ischemia–reperfusion injury constitutes a core mechanism of stroke pathology, with increased ROS production being a critical factor for mediating ischemia–reperfusion injury (Forrester et al., 2018). Ischemic stroke restricted glucose and oxygen supply, causing energy disruption and oxidative stress–induced damage primarily mediated by ROS (Qin et al., 2022). ROS production promotes the generation of pro-inflammatory cytokines and matrix metalloproteinases, exacerbating the inflammatory response (Jayaraj et al., 2019). The interplay between inflammation and oxidative stress exacerbates ischemic injury. Abhd2 overexpression significantly reduced the count of ROS^+^ monocytes. This suggests that Abhd2 may modulate biological functions of monocytes by regulating ROS expression and thereby mediate ischemic injury repair. ROS represents a pivotal target in stroke pathology. Leveraging multifunctional nanoparticles alongside a stepwise targeted drug delivery strategy facilitates the elimination of ROS in the brain injury region, thereby reducing cellular apoptosis, promoting blood–brain barrier recovery, and ameliorating rat brain damage induced by MCAO/reperfusion (Li et al., 2024). Our study has established the groundwork to investigate the monocyte-specific effects of ROS following a stroke and formulate targeted ROS clearance strategies. Furthermore, ABHD2 enrichment in the FC gamma R-mediated phagocytic pathway was observed. ABHD2 may regulate the phagocytic ability of monocytes/macrophages and thus influence stroke outcomes. Phagocytic function in stroke recovery exhibits dual characteristics as it can clear damaged cells and create conditions for recovery, while excessive phagocytosis can impair surviving neurons (Ziqing et al., 2023). Monocytes demonstrate stronger phagocytic activity than microglia at 72 hours after stroke (Ritzel et al., 2015), emphasizing that balancing monocyte phagocytic activity is crucial for stroke recovery. Upregulation of Abhd2 expression enhanced the phagocytic activity of monocytes after OGD treatment, confirming the regulatory capacity of Abhd2 on monocyte phagocytic activity. By modulating the phagocytic capacity of monocytes to maintain appropriate phagocytic activity, a potential pathway to salvage the ischemic penumbra and promote stroke recovery was established. Current studies have shown that by inhibiting complement activation, reduces microglial cell phagocytosis of surviving neurons is reduced, which can improve chronic recovery post-stroke (Alawieh et al., 2018).

By integrating detailed cellular-level analysis with traditional transcriptomic approaches, our research significantly advances understanding of the complex pathological processes underlying ischemic stroke. Our study offers valuable insights into potential novel therapeutic strategies targeting the monocyte regulatory network. Our analysis of key monocyte-associated genes and their correlations with immunity, inflammation pathways, disease genes, and CD274 reveals their profound involvement in stroke pathogenesis. It is noteworthy that we have established significant roles of key genes in ischemic stroke, validating their expression changes post-MCAO, and investigated the impact of Abhd2 on the biological functions of monocytes under conditions of glucose and oxygen deprivation. Our experimental validation strongly supports our bioinformatics predictions. Investigation of regulatory networks involving miRNAs and transcription factors further determines the molecular mechanisms underlying the functions of these key genes. Our findings highlight the dynamic changes in the immune landscape of the brain post-stroke, revealing involvement of peripheral monocytes and potential regulatory mechanisms. However, the temporal dynamics and coordination of the phagocytic and pro-inflammatory functions of monocytes have distinct implications for stroke outcomes, an aspect that warrants further investigation. While the precise role of monocytes in stroke recovery remains to be fully elucidated, our findings underscore the importance of further research into monocyte contributions in stroke therapy to unlock the potential of monocytes in stroke therapy. This study provides a robust theoretical foundation for understanding ischemic stroke mechanisms and offers novel perspectives for developing targeted treatments.

## Limitations

We have analyzed the association between key genes and stroke-related genes. To further determine the importance of these key genes in stroke pathogenesis and provide valuable insights into the role of monocytes in ischemic stroke, this study has some limitations that should be noted. It is noteworthy that our findings are limited in their clinical transformation and application of predictive models due to the lack of external validation with independent clinical datasets. The research therefore requires validation in larger patient cohorts. First, clear inclusion and exclusion criteria for study subjects need to be established to balance potential confounding factors such as age, gender, ethnicity, hypertension, and diabetes. Second, standardized protocols for peripheral blood sampling and key gene expression analysis need to be developed. The efficacy of a predictive model in diagnosing and predicting outcomes in ischemic stroke needs evaluation in large-scale, long-term clinical studies. Third, refining the scope of model application, adjusting for confounding factors, conducting stratified analyses, and exploring the diagnostic or prognostic value of the model in patients of different ages or stages of stroke are essential. Multicenter studies can assess the reliability and generalizability of model predictions, providing valuable insights into the significance of key genes in the pathophysiology of stroke. Merely assessing the impact of MCAO treatment on the gene expression profile in peripheral monocytes and conducting *in vitro* experiments is insufficient to establish a causal relationship between key genes and the pathophysiology of ischemic stroke. This limitation may have hindered our comprehensive understanding of the precise mechanisms by which key genes operate in stroke, potentially impacting the development of treatment strategies. To address this, future studies will use gene-edited mice, and specifically, develop monocyte-specific Abhd2 knockout and overexpression models to establish causal relationships between key genes and stroke outcomes. These studies would include comprehensive functional assessments through water maze tasks, rotarod tests, and histological analyses to determine the effects on infarct volume, neuroinflammation, and long-term recovery. Furthermore, our study did not differentiate between infiltrating monocytes and resident monocytes at the single-cell level, which could provide better understanding of their similarities and differences in ischemic stroke. The impact of aging on stroke was also not explored in this study. These limitations may have hindered our in-depth understanding of the specific roles of monocytes from different sources in ischemic stroke and their crosstalk mechanisms, thereby impacting a comprehensive comprehension of stroke immunoreactivity. Future investigations into the modulation of monocyte function–behavior patterns by key genes and the refinement of monocyte subpopulation roles could reveal their specific contributions to inflammation responses, immune infiltration, injury repair, and cell senescence. This would thereby lay the theoretical groundwork for developing more targeted therapeutic strategies.

## Conclusions

This integrative study of single-cell and transcriptomic data unveils the complex role of monocytes in ischemic stroke pathophysiology. In the context of ischemic stroke, enhanced communication was observed between monocytes, endothelial cells, and macrophages. Monocytes and macrophages were identified as the key cells regulating a stroke-specific SPP1 signaling network. We identified a novel seven-gene signature (SERPINH1, TCF4, ABHD2, PLAC8, GNGT2, CDH5, and SLC22A8) in stroke-associated monocytes, which underpinned a robust predictive model with high diagnostic efficacy. *In vivo* experiments validated the accuracy of the model predictions, showing significant changes in key genes within peripheral monocytes of MCAO mice. Subsequently, in-depth investigation of Abhd2 at the cellular level showed that Abhd2 overexpression enhanced the proliferation and phagocytic capacity of monocytes under OGD conditions while reducing the proportion of ROS^+^ cells. Further analysis found that these genes are intricately linked to critical immune–inflammatory pathways, including the unfolded protein response, complement activation, and interferon signaling. We also uncovered regulatory mechanisms involving miRNA networks and transcription factors, with NR4A2 emerging as a key regulator. These findings demonstrate the intricate crosstalk between central and peripheral monocytes in stroke, providing new insights into stroke pathogenesis. Importantly, our research identifies promising targets for immunotherapy and lays the foundation for developing novel, targeted approaches to stroke management, potentially advancing stroke diagnosis and treatment strategies.

## Additional files:

***Additional Table 1:***

***Additional Figure 1:*** *Identification of cell types.*

Additional Figure 1 Identification of cell types.
(A) UMAP plot showing the cell clusters revealed in this work. (B) Proportional histogram depicting the alterations in cell type proportions in ischemic stroke. (C) UMAP plot visualizing cell clusters in control and MCAO (4-h, 1-d, 2-d, 3-d, and 7-d post-ischemic stroke) mouse brains. (D) Proportional histogram showing alterations in the proportions of different cell types between the control and MCAO groups. (E) Line graphs illustrating the proportion of monocytes by location. MCAO: Middle cerebral artery occlusion; UMAP: uniform manifold approximation and projection.

***Additional Figure 2:*** *Cellular communication analysis indicating the key roles of monocytes in the SPP1 and TNF signaling pathways.*

Additional Figure 2 Cellular communication analysis indicating the key roles of monocytes in the SPP1 and TNF signaling pathways.
Heatmap depicting the interactions among cell types in the SPP1 (A) and TNF (B) signaling pathways. Circle plot visualizing the cell–cell communication between different groups of sham and MCAO samples in the SPP1 (C) and TNF (D) signaling pathways. Heatmap displaying the network centrality scores of the SPP1 (E) and TNF (F) signaling pathways. Visualizing the contribution of each ligand–receptor pair to the SPP1 (G) and TNF (H) signaling pathways. (I) Bubble plot depicting all the significant interactions (ligand–receptor pairs) associated with the SPP1 and TNF signaling pathways. (J) Performance evaluation of the predictive model by ROC curves in the expanded validation set. MCAO: Middle cerebral artery occlusion; ROC: receiver operating characteristic; SPP1: osteopontin; TNF: tumor necrosis factor.

## Data Availability

*All relevant data are within the paper and its Additional files*.
