# Supplementary figures and images for: Decoding monocyte signatures in ischemic stroke: A multi-scale transcriptomic approach
Source: Neural Regen Res. 2025 Sep 3;21(7):3209–24. doi: 10.4103/NRR.NRR-D-24-01669 (PMC13378958; doi:10.4103/NRR.NRR-D-24-01669)

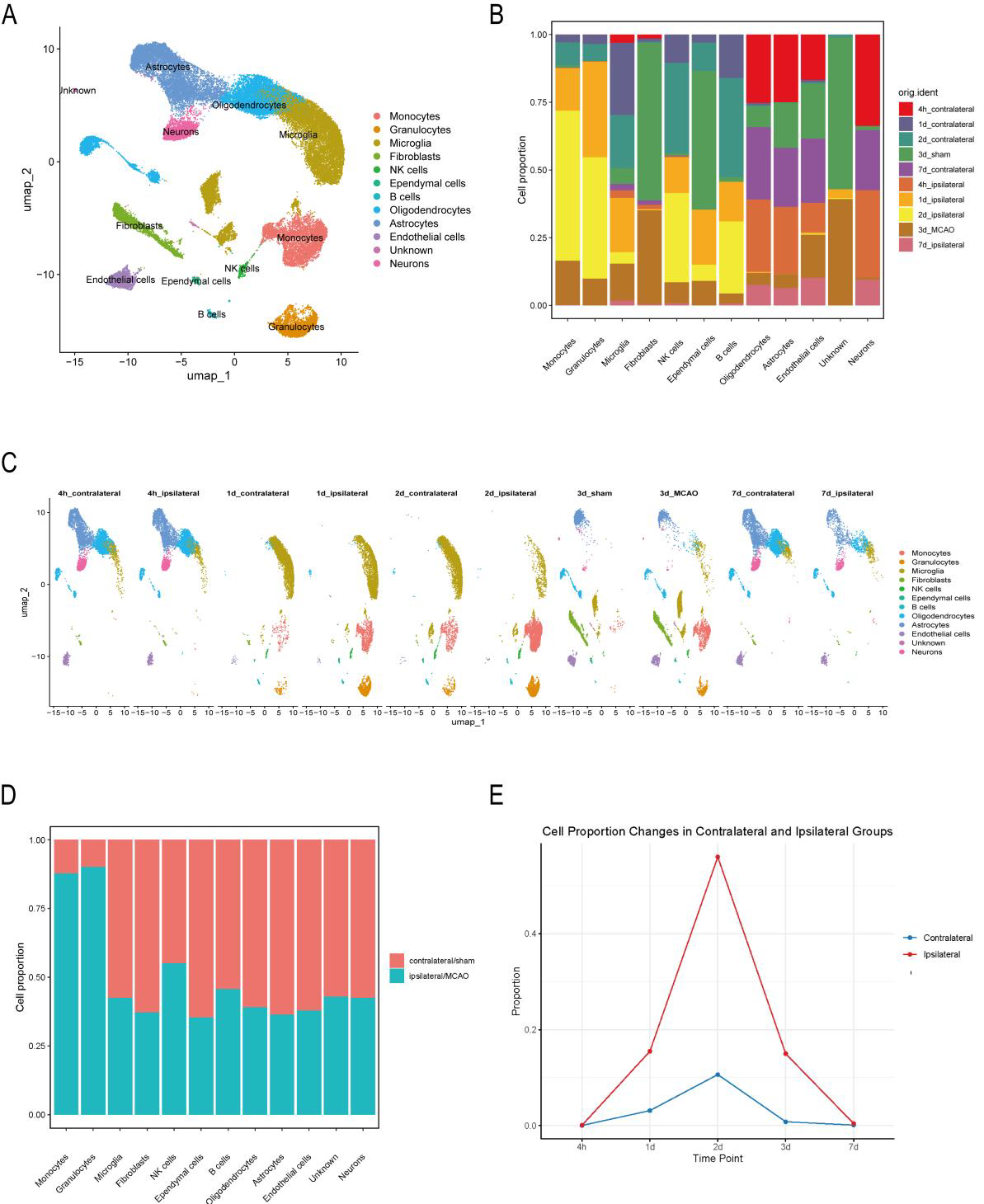

Supplement: Supplementary file 1 [file NRR-21-3209_Suppl1.tif]

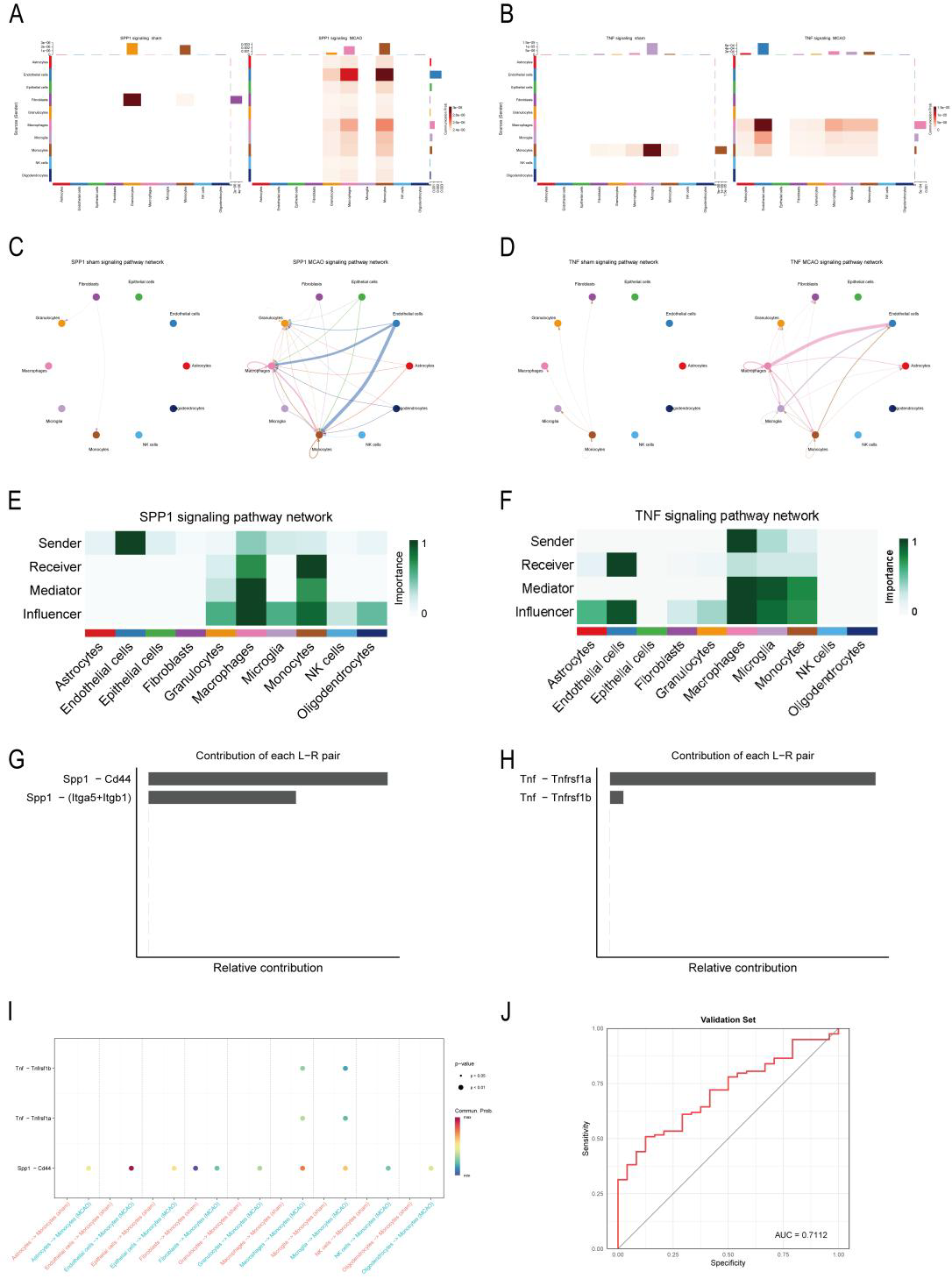

Supplement: Supplementary file 2 [file NRR-21-3209_Suppl2.tif]
